# Supplementary material for: Cost-utility analysis of LEGO based therapy for school children and young people with autism spectrum disorder: results from a randomised controlled trial
Source: BMJ Open. 2022 Jan 17;12(1):e056347. doi: 10.1136/bmjopen-2021-056347 (PMC8765033; doi:10.1136/bmjopen-2021-056347)
Supplement: Supplementary data [file bmjopen-2021-056347supp001.pdf]

Appendix 1: Inclusion and exclusion criteria

Inclusion criteria:

A participant was included if the CYP:

- Was aged between 7 and 15 years at the time of randomisation of the school.
- Attended a mainstream school in years two to 10.
- The CYP and parent/guardian had a sufficient understanding of English to be able to provide informed assent/informed consent (as appropriate) and read the LEGO®-based therapy instructions.
- Had an ASD clinical diagnosis from a qualified assessing clinician or team [based on best-practice guidance leading to an ICD-10 or DSM-5 diagnosis as reported by the CYP's parent/guardian and in the CYP's school records (this may have included the school's special educational needs (SEN) register, an individual education plan (IEP), individual health care plan, my support plan (MSPs), education health care plans (EHCPs), individual learning plans (ILP's) or equivalent).
- Had the ability to follow and understand simple instructions (as determined by the associated teacher/TA or parent/guardian).
- Scored 15 or higher on the Social Communication Questionnaire.

A school was included if:

- It was a mainstream school located in Leeds, York, Sheffield or surrounding areas in the North of England. This excludes specialist and independent schools.
- It had not used LEGO®-based therapy with the CYP in the current or preceding school term. For research purposes, LEGO®-based therapy was defined as meeting all of the main fidelity checklist criteria.
- They had at least one CYP diagnosed with ASD (in line with CYP inclusion criteria above)

Exclusion criteria:

A participant (CYP) was not eligible to take part in the study if:

- They had physical impairments which would prevent them participating in the activities (as assessed by the associated teacher/TA).

Wang H-I, *et al. BMJ Open* 2022; 12:e056347. doi: 10.1136/bmjopen-2021-056347

Appendix 2: Flow diagram

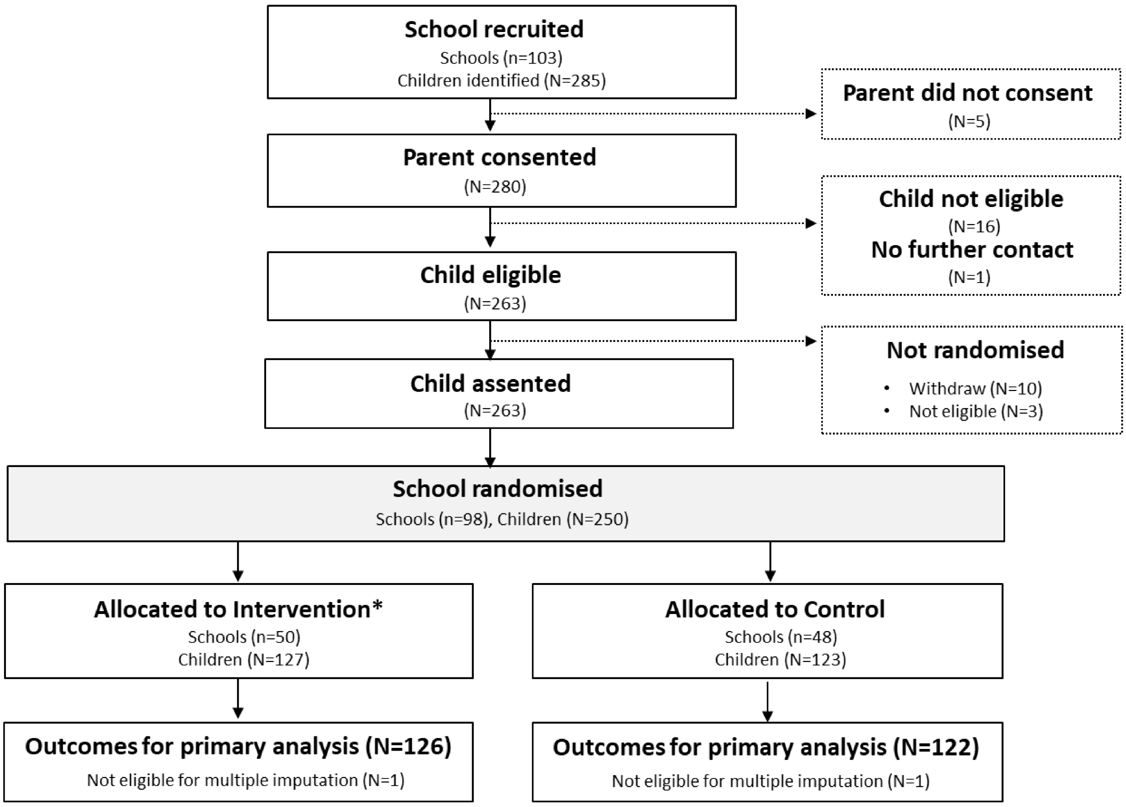

*following ITT principles, school (n=1) children (N-3) allocated to control, but received intervention are included in the control arm

Appendix 3: Intervention costs by trial arm

	**Total cost (£)**	**Cost per session per child (£)**
Training costs		
Trainer fee	£4,262	£2.01
Refreshment costs	£10	£0.00
Consumable costs	£178	£0.08
Trainer’s travel costs	£740	£0.35
Total	£5,685	£2.45
Intervention costs		
LEGO	£3,903	£1.84
Intervention	£4,027	£1.90
Additional help	£77.3	£0.04
Consumables	£580.5	£0.27
Total	£8,587.8	£4.05

Wang H-I, *et al. BMJ Open* 2022; 12:e056347. doi: 10.1136/bmjopen-2021-056347

Appendix 4: Average service use by trial arm (complete case, n=139)

		Baseline		0-20 weeks		20-52 weeks	
	Unit	LEGO®-based therapy, N=80 Mean (sd)	Usual care, N=59 Mean (sd)	LEGO®-based therapy. N=80 Mean (sd)	Usual support N=59 Mean (sd)	LEGO®-based therapy, N=80 Mean (sd)	Usual support N=59 Mean (sd)
**NHS and PSS**							
Community-based services							
CAMHS related	Session	0.70 (2.76)	0.31 (0.88)	0.23 (0.88)	0.36 (1.06)	0.63 (2.81)	0.54 (2.46)
Non-CAMHS related							
GP	Appointment	0.45 (0.97)	0.24 (0.50)	0.39 (1.02)	0.22 (0.49)	0.71 (1.41)	0.66 (0.96)
Allied health professionals	Appointment	0.44 (0.93)	0.68 (2.20)	0.43 (1.00)	0.34 (0.71)	0.78 (1.96)	0.69 (1.56)
Social care services	Appointment	0.49 (1.65)	0.47 (1.34)	0.26 (1.00)	0.39 (2.11)	0.99 (2.62)	0.80 (2.06)
Hospital-based services / acute services							
Emergency services	Visit	0.19 (0.80)	0.12 (0.46)	0.13 (0.43)	0.03 (0.26)	0.20 (0.60)	0.17 (0.62)
Inpatient stay							
Mental health related	Night	-	-	-	-	-	-
Non-mental health related	Night	0.01 (0.11)	-	0.01 (0.11)	-	-	-
Outpatient visit / day case							
Mental health related	Visit	0.06 (0.37)	0.10 (0.44)	0.08 (0.38)	0.07 (0.31)	0.05 (0.22)	0.29 (1.37)
Non-mental health related	Visit	0.31 (1.71)	0.17 (0.59)	0.19 (0.75)	0.24 (0.63)	0.09 (0.40)	0.24 (0.95)
Medication							
Mental health related	Type	0.36 (0.80)	0.20 (0.48)	0.43 (0.81)	0.27 (0.55)	0.48 (0.87)	0.32 (0.51)
Non-mental health related	Type	0.33 (0.62)	0.41 (0.93)	0.41 (0.90)	0.53 (1.33)	0.38 (0.79)	0.59 (1.23)
**Education system related**							

School-based health	Hour	2.60 (10.00)	2.17 (6.94)	1.40 (5.80)	3.12 (10.51)	8.80 (32.99)	2.17 (10.06)
General support*	Hour	8.54 (7.26)	7.93 (12.68)	7.48 (9.25)	9.37 (17.34)	18.94 (30.32)	27.32 (45.13)
Intervention support*	Hour	5.50 (10.50)	4.66 (6.74)	6.78 (8.28)	7.15 (11.03)	8.37 (7.97)	7.72 (10.56)
**Private expanses – out of pocket**							
Childcare	Session	3.05 (7.35)	4.10 (11.05)	4.14 (9.30)	5.98 (12.37)	5.86 (13.58)	12.29 (25.20)
**Productivity**							
Parental productivity	Day	0.45 (1.18)	0.31 (0.93)	0.45 (1.04)	0.52 (1.51)	0.58 (1.13)	0.59 (1.40)

CAMHS: Child and Adolescent Mental Health Services, including child psychiatrist, child psychotherapist, child psychologist, clinical psychologist, mental health nurse, family therapist, and Primary mental health worker (PMHW)

Allied health professionals included community nurse, community paediatrician, occupational therapist, physiotherapist, and Speech and Language therapist

Social care services included social care worker, home care worker, family support worker, drug and alcohol support worker, and Helpline (e.g. Samaritans)

Childcare included paid childcare, after school club, religious club, and special clubs for autism children

*based on 117 teacher-reported questionnaires (68 from I-socialise arm and 48 from usual care arm)

Wang H-I, *et al. BMJ Open* 2022; 12:e056347. doi: 10.1136/bmjopen-2021-056347

BMJ Publishing Group Limited (BMJ) disclaims all liability and responsibility arising from any reliance placed on this supplemental material which has been supplied by the author(s)

Appendix 5: EQ-5D-Y responses by trial arms by data collection time points

**Usual support (n=59)**	**Baseline**			**20 Weeks**			**52 Weeks**		
	**Level 1 n (%)**	**Level 2 n (%)**	**Level 3 n (%)**	**Level 1 n (%)**	**Level 2 n (%)**	**Level 3 n (%)**	**Level 1 n (%)**	**Level 2 n (%)**	**Level 3 n (%)**
Mobility	52 (88.1)	7 (11.9)	-	46 (78.0)	13 (22.0)	-	46 (78.0)	13 (22.0)	-
Self-care	17 (28.8)	32 (54.2)	10 (17.0)	16 (27.1)	37 (62.7)	6 (10.2)	14 (23.7)	35 (59.3)	10 (17.0)
Usual activity	25 (42.4)	29 (49.1)	5 (8.5)	25 (42.4)	27 (45.8)	7 (11.8)	22 (37.3)	30 (50.8)	7 (11.9)
Pain/discomfort	45 (76.3)	13 (22.0)	1 (1.7)	37 (62.7)	22 (37.3)	-	38 (64.4)	21 (35.6)	-
Anxiety/depression	15 (25.4)	35 (59.3)	9 (15.3)	16 (27.1)	34 (57.6)	9 (15.3)	18 (30.5)	26 (44.1)	15 (25.4)
**Lego-based therapy (n=80)**	**Baseline**			**20 Weeks**			**52 Weeks**		
	**Level 1 n (%)**	**Level 2 n (%)**	**Level 3 n (%)**	**Level 1 n (%)**	**Level 2 n (%)**	**Level 3 n (%)**	**Level 1 n (%)**	**Level 2 n (%)**	**Level 3 n (%)**
Mobility	66 (82.5)	11 (13.7)	3 (3.8)	61 (76.2)	16 (20.0)	3 (3.8)	64 (80.0)	13 (16.2)	3 (3.8)
Self-care	25 (31.3)	36 (45.0)	19 (23.7)	26 (32.5)	42 (52.5)	12 (15.0)	29 (36.3)	36 (45.0)	15 (18.7)
Usual activity	33 (41.2)	41 (51.2)	6 (7.5)	35 (43.7)	36 (45.0)	9 (11.3)	43 (53.7)	33 (41.3)	4 (5.0)
Pain/discomfort	64 (80.0)	14 (17.5)	2 (2.5)	63 (78.7)	13 (16.3)	4 (5.0)	59 (73.8)	19 (23.7)	2 (2.5)
Anxiety/depression	40 (50.0)	33 (41.3)	7 (8.7)	37 (46.2)	34 (42.5)	9 (11.3)	39 (48.7)	29 (36.3)	12 (15.0)

*Level 1: none, Level 2: some, Level 3: extreme

Appendix 6: CHU-9D responses by trial arms by data collection time points

**Usual support (n=45)**			**Baseline**					**20 weeks**					**52 weeks**		
	**Level 1**	**Level 2**	**Level 3**	**Level 4**	**Level 5**	**Level 1**	**Level 2**	**Level 3**	**Level 4**	**Level 5**	**Level 1**	**Level 2**	**Level 3**	**Level 4**	**Level 5**
Worried	20 (44.4)	16 (35.6)	7 (15.6)	-	2 (4.4)	22 (48.9)	12 (26.7)	5 (11.1)	3 (6.7)	3 (6.7)	26 (57.8)	13 (28.9)	1 (2.2)	3 (6.7)	2 (4.4)
Sad	32 (71.1)	8 (17.8)	2 (4.4)	2 (4.4)	1 (2.2)	29 (64.4)	6 (13.3)	5 (11.1)	4 (8.9)	1 (2.2)	34 (75.6)	4 (8.9)	2 (4.4)	1 (2.2)	4 (8.9)
Annoyed	27 (60.0)	13 (28.9)	4 (8.9)	-	1 (2.2)	27 (60.0)	10 (22.2)	5 (11.1)	-	3 (6.7)	27 (60.0)	10 (22.2)	5 (11.1)	2 (4.4)	1 (2.2)
Tired	9 (20.0)	15 (33.3)	8 (17.8)	5 (11.1)	8 (17.8)	12 (26.7)	13 (28.9)	9 (20.0)	5 (11.1)	6 (13.3)	10 (22.2)	16 (35.6)	6 (13.3)	6 (13.3)	7 (15.6)
Pain	28 (62.2)	6 (13.3)	6 (13.3)	3 (6.7)	2 (4.4)	21 (46.7)	11 (24.4)	4 (8.9)	2 (4.4)	7 (15.6)	24 (53.3)	12 (26.7)	7 (15.6)	-	2 (4.4)
Sleep	21 (46.7)	12 (26.7)	5 (11.1)	3 (6.7)	4 (8.9)	20 (44.4)	3 (6.7)	7 (15.6)	9 (20.0)	6 (13.3)	17 (37.8)	16 (35.6)	4 (8.9)	4 (8.9)	4 (8.9)
Daily routine	23 (51.1)	12 (26.7)	4 (8.9)	3 (6.7)	3 (6.7)	19 (42.2)	11 (24.4)	7 (15.6)	4 (8.9)	4 (8.9)	24 (53.3)	10 (22.2)	5 (11.1)	3 (6.7)	3 (6.7)
Work	27 (60.0)	15 (33.3)	2 (4.4)	1 (2.2)	-	23 (51.1)	11 (24.4)	7 (15.6)	1 (2.2)	3 (6.7)	28 (62.2)	7 (15.6)	7 (15.6)	1 (2.2)	2 (4.4)
Able to join activities	23 (51.1)	6 (13.3)	10 (22.2)	3 (6.7)	3 (6.7)	10 (22.2)	14 (31.1)	10 (22.2)	9 (20.0)	2 (4.4)	20 (44.4)	10 (22.2)	10 (22.2)	1 (2.2)	4 (8.9)
**Lego-based therapy (n=51)**			**Baseline**					**20 weeks**					**52 weeks**		
	**Level 1**	**Level 2**	**Level 3**	**Level 4**	**Level 5**	**Level 1**	**Level 2**	**Level 3**	**Level 4**	**Level 5**	**Level 1**	**Level 2**	**Level 3**	**Level 4**	**Level 5**
Worried	34 (66.7)	8 (15.7)	3 (5.9)	2 (3.9)	4 (7.8)	33 (64.7)	6 (11.8)	7 (13.7)	1 (2.0)	4 (7.8)	34 (66.7)	7 (13.7)	6 (11.8)	3 (5.9)	1 (2.0)
Sad	40 (78.4)	5 (9.8)	2 (3.9)	1 (2.0)	3 (5.9)	37 (72.5)	5 (9.8)	5 (9.8)	1 (2.0)	3 (5.9)	36 (70.6)	6 (11.8)	6 (11.8)	2 (3.9)	1 (2.0)
Annoyed	37 (72.5)	7 (13.7)	4 (7.8)	2 (3.9)	1 (2.0)	34 (66.7)	10 (19.6)	5 (9.8)	-	2 (3.9)	37 (72.5)	8 (15.7)	3 (5.9)	1 (2.0)	2 (3.9)
Tired	14 (27.5)	19 (37.3)	5 (9.8)	3 (5.9)	10 (19.6)	17 (33.3)	19 (37.3)	4 (7.8)	7 (13.7)	4 (7.8)	13 (25.5)	19 (37.3)	6 (11.8)	4 (7.8)	9 (17.6)
Pain	33 (64.7)	9 (17.6)	3 (5.9)	1 (2.0)	5 (9.8)	28 (54.9)	9 (17.6)	7 (13.7)	3 (5.9)	4 (7.8)	27 (52.9)	13 (25.5)	6 (11.8)	3 (5.9)	2 (3.9)
Sleep	26 (51.0)	10 (19.6)	6 (11.8)	3 (5.9)	6 (11.8)	23 (45.1)	8 (15.7)	8 (15.7)	4 (7.8)	8 (15.7)	20 (39.2)	11 (21.6)	11 (21.6)	3 (5.9)	6 (11.8)
Daily routine	29 (56.9)	8 (15.7)	4 (7.8)	4 (7.8)	6 (11.8)	24 (47.1)	15 (29.4)	7 (13.7)	3 (5.9)	2 (3.9)	27 (52.9)	12 (23.5)	8 (15.7)	3 (5.9)	1 (2.0)
Work	34 (66.7)	7 (13.7)	6 (11.8)	1 (2.0)	3 (5.9)	33 (64.7)	10 (19.6)	5 (9.8)	3 (5.9)	-	32 (62.7)	11 (21.6)	4 (7.8)	2 (3.9)	2 (3.9)
Able to join activities	30 (58.8)	6 (11.8)	5 (9.8)	4 (7.8)	6 (11.8)	24 (47.1)	8 (15.7)	6 (11.8)	8 (15.7)	5 (9.8)	20 (39.2)	10 (19.6)	7 (13.7)	8 (15.7)	6 (11.8)

* Level 1: No, Level 2: A little bit, Level 3: A bit, Level 4: Quite a lot, Level 5 Very

Appendix 7: Sensitivity analyses

LEGO®-based therapy vs. usual support	Incremental costs (£), (95% CI)	Incremental QALYs (95% CI)	ICER (£/QALY gained), (95% CI)
Scenario 1: Complete case analysis from NHS perspective	-1,280 (-4,578 to 2,081)	0.011 (-0.017 to 0.040)	Dominant
Scenario 2: CUA from NHS and education perspective	-511 (-1,452 to 392)	0.009 (-0.008 to 0.028)	Dominant
Scenario 3: CUA from societal perspective	-376 (-1,377 to 595)	0.009 (-0.008, 0.028)	Dominant
Scenario 4: Assume outcomes were measured using CHU-9D	-246 (-719 to 246)	0.029 (0.009 to 0.049)	Dominant
