# Supplementary material for: Cost-utility analysis of LEGO based therapy for school children and young people with autism spectrum disorder: results from a randomised controlled trial
Source: BMJ Open. 2022 Jan 17;12(1):e056347. doi: 10.1136/bmjopen-2021-056347 (PMC8765033; doi:10.1136/bmjopen-2021-056347)
Supplement: Author's manuscript [file bmjopen-2021-056347.draft_revisions.pdf]

BMJ Open

BMJ Open is committed to open peer review. As part of this commitment we make the peer review history of every article we publish publicly available.

When an article is published we post the peer reviewers' comments and the authors' responses online. We also post the versions of the paper that were used during peer review. These are the versions that the peer review comments apply to.

The versions of the paper that follow are the versions that were submitted during the peer review process. They are not the versions of record or the final published versions. They should not be cited or distributed as the published version of this manuscript.

BMJ Open is an open access journal and the full, final, typeset and author-corrected version of record of the manuscript is available on our site with no access controls, subscription charges or pay-per-view fees (http://bmjopen.bmj.com).

If you have any questions on BMJ Open's open peer review process please email info.bmjopen@bmj.com

BMJ Open

Cost-utility analysis of LEGO® based therapy for school children and young people with autism spectrum disorder: results from a randomised controlled trial

Journal:	*BMJ Open*
Manuscript ID	bmjopen-2021-056347
Article Type:	Original research
Date Submitted by the Author:	12-Aug-2021
Complete List of Authors:	Wang, Han-I; University of York, Department of Health Sciences<br>Wright, Barry; University of York; Leeds and York Partnership NHS Foundation Trust<br>Bursnall, Matthew; The University of Sheffield, School of Health and Related Research (ScHARR)<br>Cooper, Cindy; The University of Sheffield<br>Kingsley, Ellen; Leeds and York Partnership NHS Foundation Trust, COMIC Research Team<br>Le Couteur, Ann; Newcastle University,<br>Biggs, Katie; The University of Sheffield, Clinical Trials Research Unit, ScHARR<br>McKendrick, Kirsty; The University of Sheffield, Clinical Trials Unit<br>Gomez de la Cuesta, Gina; Play Included CIC<br>Chater, Tim; The University of Sheffield, School of Health and Related Research<br>Barr, Amy; The University of Sheffield<br>Solaiman, Kiera; The University of Sheffield<br>Packham, Anna; The University of Sheffield<br>Marshall, David; University of York, Lime Trees CAMHS, Research Team<br>Varley, Danielle; University of York Department of Health Sciences<br>Nekooi, Roshanak; Leeds and York Partnership NHS Foundation Trust<br>Gilbody, Simon; University of York, Department of Health Sciences<br>Parrott, Steve; University of York
Keywords:	HEALTH ECONOMICS, Child & adolescent psychiatry < PSYCHIATRY, Clinical trials < THERAPEUTICS

SCHOLARONE™
Manuscripts

I, the Submitting Author has the right to grant and does grant on behalf of all authors of the Work (as defined in the below author licence), an exclusive licence and/or a non-exclusive licence for contributions from authors who are: i) UK Crown employees; ii) where BMJ has agreed a CC-BY licence shall apply, and/or iii) in accordance with the terms applicable for US Federal Government officers or employees acting as part of their official duties; on a worldwide, perpetual, irrevocable, royalty-free basis to BMJ Publishing Group Ltd ("BMJ") its licensees and where the relevant Journal is co-owned by BMJ to the co-owners of the Journal, to publish the Work in this journal and any other BMJ products and to exploit all rights, as set out in our licence.

The Submitting Author accepts and understands that any supply made under these terms is made by BMJ to the Submitting Author unless you are acting as an employee on behalf of your employer or a postgraduate student of an affiliated institution which is paying any applicable article publishing charge ("APC") for Open Access articles. Where the Submitting Author wishes to make the Work available on an Open Access basis (and intends to pay the relevant APC), the terms of reuse of such Open Access shall be governed by a Creative Commons licence – details of these licences and which Creative Commons licence will apply to this Work are set out in our licence referred to above.

Other than as permitted in any relevant BMJ Author's Self Archiving Policies, I confirm this Work has not been accepted for publication elsewhere, is not being considered for publication elsewhere and does not duplicate material already published. I confirm all authors consent to publication of this Work and authorise the granting of this licence.

For peer review only

Cost-utility analysis of LEGO® based therapy for school children and young people with autism spectrum disorder: results from a randomised controlled trial

Authors

Han-I Wang[1], Barry Wright [1,2], Matthew Bursnall[3], Cindy Cooper[3], Ellen Kingsley[2], Ann Le Couteur[4], Dawn Teare[3], Katie Biggs[3], Kirsty McKendrick[3], Gina Gomez de la Cuesta[5], Tim Chater[3], Amy Barr[3], Kiera Solaiman[3], Anna Packham[3], David Marshall[1], Danielle Varley[1], Roshanak Nekooi[2], Simon Gilbody[1], Steve Parrott[1]

[1]University of York, York, UK
[2]Leeds and York Partnership NHS Foundation Trust, Leeds, UK
[3]CTRU, University of Sheffield, Sheffield, UK
[4]Newcastle University, Newcastle, UK
[5]Play Included CIC

Corresponding author

Name: Dr. Han-I Wang
Email: han-i.wang@york.ac.uk
Address: Department of Health Sciences, Seebohm Rowntree Building, University of York, Heslington, York, YO10 5DD
Tel: 01904-321817

Key words: cost, effectiveness, EQ-5D-Y, LEGO, Play Brick Therapy, autism, ASD, RCT

Word count: 3838/4000

For peer review only

For peer review only - http://bmjopen.bmj.com/site/about/guidelines.xhtml

Abstract

Objectives: To assess the cost-effectiveness of LEGO® based therapy compared to usual support.

Design: cost-utility analysis alongside randomised control trial.

Setting: mainstream primary and secondary schools in the UK.

Participants: 248 children and young people (CYP) with autism spectrum disorder (ASD) aged 7-15 years.

Intervention: LEGO® based therapy is a group social skills intervention designed specifically for CYP with ASD. Through play, CYP learn to use the skills such as joint attention, sharing, communication, and group problem solving. CYP randomised to the intervention arm received 12 weekly sessions of LEGO® based therapy and usual support, while CYP allocated to control arm received usual support only.

Main outcome measures: Average costs based on NHS and personal social services perspective and quality adjusted life years (QALYs) measured by EQ-5D-Y over time horizon of one year were collected during the trial. Incremental cost-effectiveness ratio (ICER) was calculated, and non-parametric bootstrapping was conducted. The uncertainty around the ICER estimates was presented using cost-effectiveness acceptability curve (CEAC). A set of sensitivity analyses were conducted to assess the robustness of the primary findings.

Results: After adjustment and bootstrapping, on average, CYP in LEGO® based therapy group incurred less costs (incremental cost was -£251 (95% CI -£752 to £268)) and gained marginally improvement in QALYs (QALYs gained 0.009 (95% CI −0.008 to 0.028)). The CEAC shows that the probability of LEGO® based therapy being cost-effective was 94% at the WTP threshold of £20,000 per QALY gained. Results of sensitivity analyses were consistent with the primary outcomes.

Conclusion: Compared to usual support, LEGO® based therapy produced marginal reduction in costs and improvement in QALYs. Results from both primary and sensitivity analyses suggested that LEGO® based therapy was likely to be cost-effective.

Trial registration: ISRCTN64852382

For peer review only

Strengths and limitations of this study

1. The first economic evaluation study of LEGO® based therapy.
2. The data are from a large sample size trial in ASD, and the method followed published best-practice guidance.
3. The study accounts for the costs measured from a range of perspectives and the QALYs measured by different instruments.
4. Resource use data were collected retrospectively and may be affected by inaccurate recall.
5. Further model-based evaluation is required to assess long term cost-effectiveness of LEGO® based therapy.

For peer review only

For peer review only - http://bmjopen.bmj.com/site/about/guidelines.xhtml

1. Background

Autism spectrum disorders (ASDs) are a group of lifelong developmental conditions defined by impairments in social interactions, communication skills, and presence of restricted and stereotypical behaviours (1). It is estimated that around 120,000 children and young people (CYP)(1.1% of total CYP) in the UK have a diagnosis of ASD (2–4). CYP with ASD have differing health and quality of life outcomes to neurotypically developing people and over their lifetime this is likely to have a financial impact on their families or carers. In the UK, the annual cost of supporting children with ASD has been estimated at between £3.1 and £3.4 billion (in 2011 value) with higher values when there is associated intellectual disability. The main cost driver of the annual support cost was special education (47%) followed by parental productivity loss (12%). Medical services accounted for only 4 % of the total (5).

Due to the substantial financial burden borne by the health care system, education system, and families of CYP with ASD, the Lancet Psychiatry Commission (LPC) emphasizes the need to not only focus on the effectiveness of mental health services, but also on their economic benefits (6). However, with the evolving intervention landscape of ASD, only two economic evaluation studies were found for CYP with ASD (7). One investigated the cost-effectiveness of a communication intervention (8) and another investigated an early intervention programme (9). These preliminary studies suggested that early intervention for children with suspected ASD was cost-effective, but communication-focused therapy for preschool children with ASD was not, with more research being recommended.

LEGO® based therapy (10) is a group based social skills intervention specifically designed for CYP with ASD which does not rely on adult-led teaching of skills. It has become very popular in the UK with many local authorities now recommending its use in schools (11). Despite the growing interest and substantial economic burden of ASD, no economic evaluation study has been done for LEGO® based therapy. Hence, the aim of this study was to access the cost-effectiveness of LEGO® based therapy. This paper reports the economic evaluation results of LEGO® based therapy for CYP with ASD alongside the Investigating SOcial Competence and Isolation in children with Autism taking part in LEGO® based therapy clubs In School Environments (I-SOCIALISE) trial (12).

2. Methods

2.1 Trial design and participants

This economic evaluation was embedded in the I-SOCIALISE trial, a multisite, pragmatic, two-arm, school-level cluster randomised controlled trial (RCT) for CYP with ASD. Details of

For peer review only - http://bmjopen.bmj.com/site/about/guidelines.xhtml

I-SOCIALISE trial have been published elsewhere (12). In short, CYP between the ages of seven and 15 years with a diagnosis of ASD were recruited from mainstream primary and secondary schools in the North of England between October 2018 and May 2019. To be eligible, CYP needed to have a diagnosis on the autism spectrum and score 15 or higher on the Social Communication Questionnaire (SCQ) and to be able to follow and understand simple instructions and so engage in the groups. CYP in schools that were allocated by remote randomisation to the intervention arm received 12 sessions of LEGO® based therapy in school for one hour per week. There was some flexibility in weekly delivery to accommodate school timetables. They also received usual support from school, GPs and any other professionals. CYP in schools allocated to the control arm received usual support only. Informed consent and baseline measurements were obtained and completed prior to school randomisation. All CYP were followed up 20 and 52 weeks after randomisation and completed further outcome measures. A flowchart of the study can be found in Appendix 1.

2.2 Intervention

LEGO® based therapy is a group social skills intervention designed specifically for CYP with social communication difficulties such as ASD. CYP build LEGO® models together in small groups with light facilitation by a trained adult (13,14). The CYP work together taking on one of three roles: the engineer, who reads the LEGO® set instructions; the supplier, who finds the correct pieces according to the instructions from the engineer; and the builder, who builds the model with the pieces from the supplier and the instructions from the engineer. Key elements of the intervention include the use of CYP-led collaborative building and learning through play which promote the learning and use of such skills as joint attention, sharing, communication, and group problem solving. The trained adult takes a guiding role rather than an explicitly directive one, allowing the CYP to work together and solve their own challenges. Group rules and rewards are used to foster motivation and engagement in social interactions.

2.3 Outcomes

The health outcomes for the current study were quality adjusted life years (QALYs) measured by the EQ-5D-Y (proxy version) (15) and the Child Health Utility 9D (CHU-9D) (16). EQ-5D-Y (proxy version) is a five-item questionnaire that allows a proxy person (i.e. parent/guardian) to complete the measure for CYP. The EQ-5D-Y instrument comprises five dimensions (mobility, looking after themselves, doing usual activities, having any pain or discomfort, and feeling worried or sad) and has been shown to be a reliable and valid instrument for use in CYP and adolescents (15). The CHU-9D is a CYP-completed nine-item questionnaire comprising nine dimensions: worried, sad, pain, tired, annoyed, schoolwork/homework, sleep, daily routine, and able to join in activities (16).

For peer review only - http://bmjopen.bmj.com/site/about/guidelines.xhtml

Individual-level responses to EQ-5D-Y and CHU-9D were used to estimate utilities based on UK population valuation sets (17,18). A utility represents a CYP's health state and is constrained between 0 and 1, where 0 refers to death and 1 perfect health. The estimated utilities at baseline and follow-up were further joint using the area under the curve approach (19) to calculate QALYs.

2.4 cost measurement

Two cost perspectives were considered in this study: 1) a NHS and personal social service (NHS/PSS) perspective, which included costs related to healthcare and social services, and 2) a societal perspective, which additionally included costs of education-related services, parental out-of pocket expenses (such as childcare and private courses), and parental productivity costs (time off work due to child's autism).

Cost of the intervention

Cost of the intervention included the cost of training and the cost of delivering LEGO® based therapy. Training costs were measured by the time spent by the trainer and included travel costs and the cost of materials used in the training. Costs associated with delivering the LEGO® based therapy were measured based on the time spent by facilitators to plan and conduct sessions and included relevant overhead costs. All relevant data were collected using the tailored questionnaires completed by the study team and therapists.

Cost of the service use

Service use was collected using the tailored questionnaires (completed by the parent/guardian and separately by an associated teacher of each CYP in the study who knew the CYP well), which was originally developed based on Barrett's study (20) and has been successfully adapted for use in school-based trials (21,22). The parent/guardian-completed questionnaire captured data on the use of health and social services, school-based services (including school-based health services, general and intervention support), parental private expenses, and parental productivity costs. Teacher-completed questionnaires captured any school-based interventions/support and the implications of a CYP's behaviour on school resource.

Service use was multiplied by unit costs to arrive at total cost in each arm. Unit costs of health and social service use were obtained from published sources (i.e. Reference Cost 2018 (23), Personal Social Services Research Unit 2018 (24), and Prescription Cost Analysis – England 2018 (25)), national survey (i.e. Childcare Costs Survey 2018 (26)), and government departments (i.e. Department for Education 2018 (27) and Green Book 2018 (28)). Privately

For peer review only - http://bmjopen.bmj.com/site/about/guidelines.xhtml

paid services were separately estimated via market prices, while productivity losses were calculated using the human capital approach, which involves multiplying time off work by UK average salary (29).

All the costs were expressed in 2018 UK sterling. Discounting of costs and QALYs was not applied, as the study time horizon was one year.

2.5 Missing data

All eligible CYPs who had both utility and cost data at any time point were referred to as complete cases. While, the eligible CYPs who had missing utility and cost data but had complete baseline assessments were referred to as base case. The identified missing utility and cost data were imputed using multiple imputation method via chained equations (30). Imputation was based on trial arm, age, gender, study site, and utility scores and Social Communication Questionnaire (SCQ) scores at baseline.

2.6 Statistical and economic analyses

The primary analysis was to calculate incremental cost-effectiveness ratio (ICER) based on the costs from NHS/PSS perspective and the QALYs measured by EQ-5D-Y. To account for uncertainty around ICER and imbalanced utility and costs at baseline, seemingly unrelated regression equations (SURE) that controlled for baseline utility (31), costs, age, gender, and SCQ scores were bootstrapped 5,000 times. The SURE approach was recommended by Glick and colleagues (19), which considers the distribution of the dependent variable and any correlation found between cost and QALY outcomes. While non-parametric bootstrap re-sampling method was suggested by Briggs and colleagues (32), as the distribution of regression residuals was likely to be skewed (33). The 5,000 bootstrapped iterations were represented graphically on the cost-effectiveness plane (CE-plane), and the cost-effectiveness acceptability curve (CEAC) was generated by plotting the probability of the intervention being cost-effective over a range of willingness-to-pay (WTP) thresholds (34). The calculated ICERs were against the national WTP threshold of £20,000-£30,000 per QALY gained to decide whether the LEGO® based therapy is cost-effectiveness (35).

To assess the robustness of our findings, a set of sensitivity analyses were conducted. First, a cost-utility analysis (CUA) (34) using complete cases was conducted to assess the impact of the missing data. Second, a CUA was performed from a NHS/PSS and education perspective to account for the economic impact from the education system. Third, a CUA was performed from a societal perspective to account for all the economic impact outside the NHS/PSS perspective, including parental productivity costs. Finally, a CUA that used the CHU-9D instead of the EQ-5D-Y to estimate QALYs based on the UK population tariff (18)

For peer review only - http://bmjopen.bmj.com/site/about/guidelines.xhtml

was conducted to assess the impact of outcome measurement instrument.

All analyses were performed on an intention-to-treat basis using Stata version 16 ((StataCorp, College Station, Texas, USA).

2.7 Ethical approval and informed consent

This study was funded by the National Institute for Health Research (NIHR) Public Health Research (PHR) programme (PHR15/49/32), and the International Standard Randomised Controlled Trial Number is ISRCTN64852382 (2). Positive ethical opinion has been obtained via the University of York Research Ethics Committee (18/HRA/0101), and the written informed consent was obtained from parents on behalf of their child. Children assented to be part of the groups and did not proceed if they were not willing.

2.8 Patient and Public Involvement

No patient involved.

3. Results

3.1 Participants

A total of 284 CYP with ASD were recruited in the trial. After removing 34 ineligible CYP and 2 CYP who were not eligible for multiple imputation due to missing baseline utilities, 248 CYP with ASD were available for primary analysis (126 were allocated to LEGO® based therapy and 122 to usual support). This sample constitutes the base-case group. On the other hand, only 139 (56.0%) CYP had both EQ-5D and resource use (from the NHS and PSS perspective) data at all three data collection time points. This sample constitutes the complete-case group.

Descriptive statistics of CYP's baseline characteristics for both complete-case and base-case are presented in Table 1. As shown, more than third-quarters of the CYP in the LEGO® based therapy and the usual care arms were male, and more than 50% of the CYP in both arms were in primary school age (ranging from 7 to 11 years old). Differences in the SCQ scores at the baseline were marginal across arms and samples. Overall, the baseline characteristics are consistent across samples (base-case and complete-case).

Table 1: Baseline characteristics by trial arm

	Base case (n=248)		Complete case (n=139)	
**Baseline characteristics**	**LEGO® based therapy (N=126)**	**Usual support (N=122)**	**LEGO® based therapy (N=80)**	**Usual support (N=59)**
Gender, n (%)				

For peer review only - http://bmjopen.bmj.com/site/about/guidelines.xhtml

Male	101 (80.2%)	91 (74.6%)	68 (85.0%)	43 (72.9%)
Age (years), n (%)				
7-11	83 (65.9%)	79 (64.8%)	54 (67.5%)	43 (72.9%)
11-15	43 (34.1%)	43 (35.2%)	26 (32.5%)	16 (27.1%)
Mean (sd)	9.7 (2.3)	9.8 (2.2)	9.6 (2.2)	9.6 (2.2)
Year from diagnosis				
Mean (sd)	3.4 (2.7)	3.6 (2.8)	3.2 (2.4)	3.6 (3.0)
Social Communication Questionnaire (SCQ) scores				
Mean (sd)	25.1 (5.2)	24.2 (5.2)	24.9 (5.1)	24.1 (5.0)
EQ-5D				
Mean (sd)	0.79 (0.11)	0.76 (0.11)	0.79 (0.12)	0.77 (0.11)
Site, n (%)				
Leeds	37 (29.4%)	38 (31.2%)	31 (38.8%)	18 (30.5%)
Sheffield	70 (55.6%)	67 (54.9%)	34 (42.5%)	31 (52.5%)
York	19 (15.1%)	17 (13.9%)	15 (18.7%)	10 (17.0%)
Number of intervention sessions				
Mean (sd)	10.3 (2.3)	-	10.5 (2.2)	-

3.2 Costs

On average, the estimated intervention cost per session per CYP was £6.5 (£2.45 for training and £4.05 for intervention delivery). The main cost driver of training costs was trainer fees, while the main cost drivers of intervention delivery costs were the costs for preparation and delivery the intervention, and the costs for LEGO® materials (Appendix 2).

In terms of service costs, the average total service costs over 52 weeks to the NHS providers (after imputation) were £524 (95%CI: £428 to 808) for the LEGO® based therapy arm compared with £678 (95% CI: £427 to 928) for the usual care arm. The cost difference is larger when the societal perspective was considered, as CYP in the LEGO® based therapy arm incurred less costs across all the perspectives. The largest cost differences occurred in education related services followed by healthcare and social services. It is worth noting that some of cost differences were likely to have been driven by high-cost cases. For instance, in complete-case, higher average cost of Child and Adolescent Mental Health Services (CAMHS)-related services in the usual care arm was driven by two high-cost cases, and higher average cost of school-based health related services in the LEGO® based therapy arm was driven by one high-cost case. These high-cost cases were kept in the analysis, as they were plausible. However, due to the high-cost cases, the cost differences need to be interpreted with caution. A more detailed overview on the service costs over 52 weeks and the resource use are presented in Table 2 and Appendix 3, respectively.

For peer review only
For peer review only - http://bmjopen.bmj.com/site/about/guidelines.xhtml

Table 2: Average costs of service use in one year by trial arm

	Base case		Complete case	
	LEGO® based therapy (n=126), £ (95% CI)	Usual support (n=122), £ (95% CI)	LEGO® based therapy (n=80), £ (95% CI)	Usual support (n=59), £ (95% cI)
**NHS and PSS**	**524 (372, 675)**	**678 (427, 928)**	**618 (428, 808)**	**752 (420, 1,083)**
Community-based services				
CAMHS related	77 (40, 114)	233 (37, 428)	117 (50, 184)	267 (19, 516)
Non-CAMHS related	115 (79, 151)	99 (69, 130)	120 (78, 161)	107 (67, 148)
Hospital-based services				
Mental health related	20 (6, 33)	45 (11, 79)	19 (4, 33)	53 (-1, 107)
Non-mental health related	60 (29, 92)	86 (37, 136)	79 (30, 128)	89 (31, 147)
Medications				
Mental health related	195 (115, 275)	129 (74, 185)	211 (121, 301)	142 (75, 208)
Non-mental health related	57 (18, 97)	85 (25, 145)	73 (22, 124)	93 (19, 167)
**Education system related**	**1,204 (949, 1,458)**	**1,437 (1,082, 1,792)**	**1,388 (989, 1,787)**	**1,633 (1,041, 2,224)**
School-based health	164 (62, 267)	88 (20, 156)	182 (48, 316)	100 (13, 186)
Intervention support	712 (496, 927)	948 (645, 1,250)	793 (458, 1,128)	1,070 (615, 1,526)
General support	327 (242, 413)	401 (262, 541)	368 (245, 492)	473 (237, 709)
**Private expenses**	**211 (129, 293)**	**317 (189, 445)**	**192 (105, 280)**	**329 (171, 487)**
Childcare	211 (129, 293)	317 (189, 445)	192 (105, 280)	329 (171, 487)
**Productivity**	**95 (57, 132)**	**114 (64, 164)**	**104 (62, 146)**	**111 (53, 170)**
Parental productivity loss	95 (57, 132)	114 (64, 164)	104 (62, 146)	111 (53, 170)
**Total costs**	**2,033 (1,710, 2,357)**	**2,546 (2,087, 3,005)**	**2,278 (1,775, 2,781)**	**2,819 (2,123, 3,515)**

CAMHS: Child and Adolescent Mental Health Services, including child psychiatrist, child psychotherapist, child psychologist, clinical psychologist, mental health nurse, family therapist, and Primary mental health worker (PMHW)
Allied health professionals included community nurse, community paediatrician, occupational therapist, physiotherapist, and Speech and Language therapist
Social care services included social care worker, home care worker, family support worker, drug and alcohol support worker, and Helpline (e.g. Samaritans)
Childcare included paid childcare, after school club, religious club, and special clubs for autism children

For peer review only - http://bmjopen.bmj.com/site/about/guidelines.xhtml

For peer review only

3.3 Outcomes

Table 3 shows the mean EQ-5D-Y (3L proxy) and CHU-9D utility scores between the two arms of the trial at each time point when scores were not imputed (complete case) and when scores were imputed (base case). As shown, in both arms, there was no significant change in EQ-5D-Y or CHU-9D utility scores from baseline to 52 weeks. The fluctuations between baseline and 20 weeks, and between 20 weeks and 52 weeks, were small in both the base and the complete cases. After calculation using the area under the curve approach, it is found that the LEGO® based therapy produced marginally higher mean QALYs (0.03 QALYs) compared to the usual support regardless of the instrument used. Further details for the responses of EQ-5D and CHU-9D in each domain can be found in Appendix 4 and Appendix 5, respectively.

Table 3: Average EQ-5D-Y and CHU-9D utility scores by trial arm

	Base case		Complete case	
Time point	LEGO® based therapy (n=126), mean (95% CI)	Usual support (n=122) mean (95% CI)	LEGO® based therapy (n=80), mean (95% CI)	Usual support (n=59) mean (95% CI)
EQ-5D-Y				
Baseline	0.79 (0.77, 0.81)	0.76 (0.74, 0.79)	0.79 (0.77, 0.81)	0.77 (0.74, 0.79)
20 weeks	0.78 (0.76, 0.81)	0.76 (0.74, 0.78)	0.79 (0.76, 0.81)	0.75 (0.74, 0.79)
52 weeks	0.79 (0.76, 0.81)	0.76 (0.74, 0.79)	0.80 (0.77, 0.82)	0.75 (0.73, 0.80)
Total QALYs	0.79 (0.77, 0.80)	0.76 (0.74, 0.78)	0.79 (0.77, 0.81)	0.76 (0.74, 0.79)
CHU-9D				
Baseline	0.83 (0.80, 0.85)	0.81 (0.79, 0.84)	0.84 (0.81, 0.87)	0.83 (0.80, 0.86)
20 weeks	0.84 (0.82, 0.86)	0.80 (0.78, 0.83)	0.83 (0.80, 0.86)	0.78 (0.74, 0.82)
52 weeks	0.83 (0.81, 0.85)	0.80 (0.77, 0.83)	0.81 (0.78, 0.84)	0.81 (0.77, 0.85)
Total QALYs	0.83 (0.82, 0.85)	0.80 (0.78, 0.82)	0.82 (0.80, 0.85)	0.80 (0.76, 0.83)

3.4 Cost-utility analysis (primary analysis)

After accounting for uncertainty and unbalanced baseline utility and characteristics, on average, CYP with ASD receiving LEGO® based therapy incurred £251 (95% CI - £268 to £752) less costs from the NHS/PSS perspective and gained 0.009 (95% CI -0.008 to 0.028) extra QALYs measured by EQ-5D-Y than those having usual support. The bootstrapped ICER results are presented in Figure 1A, and the probabilities of LEGO® based therapy being cost-effective over a range of WTP threshold are presented in Figure 1B. As shown, the simulated estimates were largely below the threshold line, and the probability of LEGO® based therapy being cost-effective is 94% at the WTP threshold of £20,000. The findings

For peer review only - http://bmjopen.bmj.com/site/about/guidelines.xhtml

suggest that the LEGO® based therapy was likely to be cost-effective, although the incremental costs and QALYs were marginal.

3.5 sensitivity analysis

Results of sensitivity analyses are detailed in Appendix 6. The mean incremental cost and QALY estimates from the complete-case were along the line of the based-case scenario, yielding a negative cost per QALY gained. Sensitivity analyses using a societal perspective to measure costs and the CHU-9D to measure QALYs were also conducted. In both sensitivity analyses, the ICER pairs lay below the recommended NICE threshold (£20,000-30,000/QALY gained), and the majority of the bootstrapped estimates sat in the fourth quadrant (Figure 2), suggesting that LEGO® based therapy was likely to be cost-effective.

4. Discussion

4.1 Principal findings

To the best of our knowledge, this is the first study evaluating the cost-effectiveness of LEGO® based therapy for CYP with ASD. Compared to usual care, the LEGO® based therapy marginally decreased the service use costs and increased the QALYs from the NHS/PSS perspective. This is evident in both primary and sensitivity analyses, which considered costs derived from various perspectives and QALYs measured by different instruments.

4.2 Implications of study

The average QALYs measured by both the EQ-5D-Y (proxy version) and the CHU9D for those in the intervention arm were higher compared to the control arm. Although the differences are small, it is observed that the bootstrap estimates plotted on the CE planes for both primary and sensitivity analyses were mainly in the fourth quadrant (bottom right-hand quadrant). This indicates that after taking uncertainty into consideration and adjusting for the imbalanced baseline utility, the differences in QALYs remain positive. It is also worth noting that such differences were unaffected regardless who filled the questionnaire. Both the parent-completed (EQ-5D) and CYP-completed (CHU9D) questionnaires showed the same positive differences.

The study also shows a reduction in average total NHS/PSS costs (albeit with wide confidence intervals), particularly through attendance at Child and Adolescent Mental Health Services (CAMHS). As mentioned in section 3.2, the lowered CAMHS costs found in the LEGO® based therapy arm was more related to a small number of CYP receiving high levels of high-tariff CAMHS support in the control arm (usual support) than the intervention arm rather than a general drop across the whole group. Such a finding indicates that CYP

For peer review only - http://bmjopen.bmj.com/site/about/guidelines.xhtml

who had co-occurring emotional and behavioural problems seemed to be receiving approximately similar support from CAMHS across both trial arms, and LEGO® based therapy did not overshadow the needs of CAMHS support. It was also found that, at the baseline, CYP in LEGO® based therapy arm had higher frequency of CAMHS support compared to control arm. Given the high threshold for receiving CAMHS support, it is likely that the CYP in LEGO® based therapy arm had more severe needs at baseline. However, after the intervention, CYP in LEGO® based therapy arm had similar support from CAMHS as those in control arm (see above) suggesting some amelioration effect or a reduced need for such high level support. Based on the literature of general research on CAMHS referrals, such reduction could be because the LEGO® based therapy improves co-morbid problems of CYP and consequently leads to a reduced likelihood of referral to CAMHS (36) or stops the CYP being seen by CAMHS (37). However, it is also possible that the school-based LEGO® based therapy improves a CYP's social skills and leads to less distress or conflict and may subsequently reduce the likelihood for referral to CAMHS (38) because of reduced levels of teacher and parent/guardian concern. Research to date, however, suggests higher parental anxiety tends to reduce referral rates not increase them in many circumstances (39).

The reduction in school intervention costs was also observed in this study. One possible explanation is that CYP in a LEGO® based therapy intervention might be less likely to be put forward for other interventions (e.g., the social use of language programme (SULP) (40)). Another possible reason might include a belief by a parent/guardian that an active intervention is happening, and so for this reason taking part in another intervention at the same time is not necessary. While both possible explanations might be valid, there was evidence that schools reported a wide range of other interventions being received by CYP with ASD, including Social Stories™, visual schedules, 1:1 mentoring, and others. Whether LEGO® based therapy reduced certain type of interventions but increases other type of interventions remains unclear. Further investigations would be needed to explore this.

4.3 Strengths and limitations

This is the first study evaluating the cost-effectiveness of LEGO® based therapy and one of only a few economic evaluation studies for CYP with ASD (8,9). Such a study is important because there is a growing popularity of LEGO® based therapy in the UK. Furthermore, since detailed resource use in school was able to be collected via teacher-completed questionnaire, our study managed to capture the cost difference in school in a more granular manner and reflect the reality in school better. Additionally, our study accounts for the costs measured from a range of perspectives and the QALYs measured by different instruments. The approach not only ensures the robustness of our findings but also can help policy makers from different sectors to make informed decision. Finally, this study has a

For peer review only - http://bmjopen.bmj.com/site/about/guidelines.xhtml

large sample size compared to other similar trials in ASD (8,41) and is one of the few ASD intervention studies to date follow up to one year (8,42). This would make our results more generalizable and robust compared to the similar study with small sample size or shorter term follow-up.

Despite the strengths mentioned above, our study was subject to three limitations. Firstly, funding sources for a few types of staff was not always clear. For example, speech and language therapists can be funded by NHS, schools or local authorities. Such diversity causes difficulties when it comes to costing and reporting the results, as detailed information about funders for each member of staff involved was unavailable. Several assumptions have been made based on service locations and published guidelines (i.e. the unit costs of Health and Social Care from PSSRU 2018) for costing. Hence, the summarised cost results for different perspectives need to be treated with caution. However, both arms were treated the same way and the overall costs (from the societal perspective) should remain robust. Secondly, a small number of high but plausible values were observed in cost estimations. Although the values did not affect the cost-effectiveness conclusions (data not shown), such values can potentially bias the cost reduction results of LEGO® based therapy. Hence, care should be taken when interpreting the cost estimates. It is none-the-less important to include this real world data and be aware of this for future studies. Thirdly, the calculated intervention costs might have been underestimated. This is because several items associated with training and intervention sessions were not costed, due to data constraints. These included opportunity costs of trainee time, opportunity costs of school venue for delivering interventions, recruitment cost if intervention rolled out, and supervision costs. However, this is unlikely to have affected the results of the dominance of the intervention over usual support, as these costs are considered to be small. This is especially the case after the allocating to every CYP for every session. Further research on the exploration and measurement of the costs with considerations is desirable. Finally, our economic analysis only assessed the short-term cost-effectiveness of LEGO® based therapy between baseline and 52-week follow-up. The cost-effectiveness beyond the one year timeframe remains unknown. Further model-based evaluation is required to assess longer term cost-effectiveness (i.e. end of school age, early adulthood or lifetime) and allow for the measurement of CYP's lost productivity in adulthood.

4.4 Future work

Our study measured the short-term cost-effectiveness of LEGO® based therapy on CYP with ASD over one year follow-up. For the long-term cost-effectiveness of LEGO® based therapy, a modelled-based economic evaluation study would be desirable to allow life-time cost-effectiveness and children's lost productivity during adulthood to be measured.

For peer review only - http://bmjopen.bmj.com/site/about/guidelines.xhtml

5. Conclusion

This study demonstrates the potential cost-effectiveness of delivering LEGO® based therapy to CYP with ASD in mainstream school settings. The findings will be of interest to NHS health and social care providers, local authorities, families and community professionals including school staff members.

For peer review only

For peer review only - http://bmjopen.bmj.com/site/about/guidelines.xhtml

Authors' contributions

HW and SP were the trial health economists and analysed the data with HW taking the lead in writing the manuscript. BW, CC, ALC, DT, KB, GGDLC, DM, DV and SG conceived the study idea and designed the project. MB and DT were the study statisticians and involved in the analysis and interpretation of the data. EK and KM managed the trial, and TC, AB, KS, AP and RN were involved in the acquisition and management of the data. All authors contributed to data interpretation and have read and approved the final manuscript.

Competing interests

The research team was aware that the LEGO® name is a registered trademark and followed the fair use policy in regard to the LEGO® brand throughout the duration of the trial. **Gina Gomez de la Cuesta** co-authored the LEGO® based therapy manual which formed the basis of the intervention delivered in the trial. The co-authors of the manual have given us full permission to use the manual without license and to develop an abridged version. They have also stated their support for us in writing our own version and will become co-authors on any future publications. **Gina Gomez de la Cuesta** has also agreed for the team to adapt the fidelity checklist used in her previous study. **Gina Gomez de la Cuesta** is a Director of Play Included a community interest company that offers training and resources for interventions involving play bricks for children. We have provisional agreement with Jessica Kingsley Publishers who have expressed interest in publishing the abridged manual. However, we are not tied to them as a publisher. There are no other financial and/or competing interests to declare.

Funding statement

This work was supported by the NIHR under its Programme Grants for Public Health Research (PHR) (Grant Reference Number PHR15/49/32)

Data sharing statement

No additional data available. However, Stata code that used for this study is available from the corresponding author, Han-I Wang, upon reasonable request.

Acknowledgments

We gratefully thank all families participating in the study. We acknowledge invaluable support and guidance from our trial steering committee (Professor David Cottrell (University of Leeds), Karen Watson (PPI), Dr Sue Fletcher-Watson (University of Edinburgh), Dr Fiona Warren (University of Exeter), Dr Michael Morton (University of Glasgow), Alison Thompson (Leeds and York Partnership NHS Foundation Trust)), and our data monitoring and ethics committee (Dr Zoe Hoare (Bangor University), Dr David Simms (Bradford District Care NHS

For peer review only - http://bmjopen.bmj.com/site/about/guidelines.xhtml

Foundation Trust), John O'Dwyer (University of Leeds)). We also gratefully acknowledge the hard work, support and advice from the following:

PPI representatives

The Young Dynamos research advisory group, the National Autistic Society, Karen Watson (PPI TSC member), the York Youth Council and several individuals including Tina Hardman and Ann LcLaren.

Participant screening and data collection

Sarah Jacob-Eshtan, Lisa Hackney, Rebecca Hargate, Sam Bennett, Emma Sellers, Holly Taylor, Alix Smith, Katie Sutherland, Richard Campbell, Rachel Hodkinson, Megan Garside, Jane Blackwell, Pavithra Kumar, Jennifer Lomas

Administrative and clerical support

Sharon Bird, Rita Lynch, Daniel Gottschalk, Katy Harmston, Thasamia Akhtar, Louise Turner, Heather Dakin

Sponsor Study monitoring

Tahir Idrees

LEGO® based therapy expertise

Elinor Brett, Abigail Dodson

Support on data management concerns

Emily Turton

Support on ethical and governance issues.

Sinead Audsley, Stephen Holland

The authors would also like to thank Daniel LeGoff for information about LEGO® based therapy in the USA and both the Leeds and York Partnership NHS Foundation Trust and the University of York for supporting the Child Oriented Mental Health Intervention Centre (COMIC) and the University of Sheffield CTRU in taking this research from an idea to a full trial.

The research team are aware that the LEGO® name is a registered trademark and will follow the fair use policy in regard to the LEGO® brand throughout the duration of the trial. The team have been in discussion with LEGO, and they have agreed for the use of this term for the project and its outputs, but not over the longer term. After PPI work, we have paired the term LEGO® based therapy with the new term Play Brick Therapy, which we suggest is used henceforth.

Disclaimer

The views and opinions expressed by authors in this publication are those of the authors and do not necessarily reflect those of the NHS, the NIHR, NETSCC, the PHR programme or

For peer review only - http://bmjopen.bmj.com/site/about/guidelines.xhtml

the Department of Health and Social Care.

Patient consent form

Not required.

Ethical approval

Ethics approval has been obtained via the University of York Research Ethics Committee (18/HRA/0101)

For peer review only

For peer review only - http://bmjopen.bmj.com/site/about/guidelines.xhtml

Reference

1. American Psychiatric Association. Diagnostic and Statistical Manual of Mental Disorders (DSM–5). [Internet]. 5th edition. Arlington: American Psychiatric Association; 2013. Available from: https://www.psychiatry.org/psychiatrists/practice/dsm

2. Baron-Cohen S, Scott FJ, Allison C, Williams J, Bolton P, Matthews FE, et al. Prevalence of autism-spectrum conditions: UK school-based population study. Br J Psychiatry J Ment Sci. 2009 Jun;194(6):500–9.

3. Baird G, Simonoff E, Pickles A, Chandler S, Loucas T, Meldrum D, et al. Prevalence of disorders of the autism spectrum in a population cohort of children in South Thames: the Special Needs and Autism Project (SNAP). Lancet Lond Engl. 2006 Jul 15;368(9531):210–5.

4. Office for National Statistics. 2011 Census Aggregate Data. UK Data Service; 2017.

5. Buescher AVS, Cidav Z, Knapp M, Mandell DS. Costs of autism spectrum disorders in the United Kingdom and the United States. JAMA Pediatr. 2014 Aug;168(8):721–8.

6. Holmes EA, Ghaderi A, Harmer CJ, Ramchandani PG, Cuijpers P, Morrison AP, et al. The Lancet Psychiatry Commission on psychological treatments research in tomorrow's science. Lancet Psychiatry. 2018 Mar 1;5(3):237–86.

7. Sampaio F, Feldman I, Lavelle TA, Skokauskas N. The cost-effectiveness of treatments for attention deficit-hyperactivity disorder and autism spectrum disorder in children and adolescents: a systematic review. Eur Child Adolesc Psychiatry [Internet]. 2021 Mar 9 [cited 2021 Mar 26]; Available from: https://doi.org/10.1007/s00787-021-01748-z

8. Byford S, Cary M, Barrett B, Aldred CR, Charman T, Howlin P, et al. Cost-effectiveness analysis of a communication-focused therapy for pre-school children with autism: results from a randomised controlled trial. BMC Psychiatry. 2015 Dec 21;15:316.

9. Penner M, Rayar M, Bashir N, Roberts SW, Hancock-Howard RL, Coyte PC. Cost-Effectiveness Analysis Comparing Pre-diagnosis Autism Spectrum Disorder (ASD)-Targeted Intervention with Ontario's Autism Intervention Program. J Autism Dev Disord. 2015 Sep;45(9):2833–47.

10. Baron-Cohen S, Cuesta GGDL, LeGoff DB, Krauss GW. LEGO®-Based Therapy: How to build social competence through LEGO®-based Clubs for children with autism and related conditions. Jessica Kingsley Publishers; 2014. 146 p.

For peer review only

For peer review only - http://bmjopen.bmj.com/site/about/guidelines.xhtml

11. North Yorkshire County Council. North Yorkshire County Council Intervention Guidance 2019. [Internet]. 2019 [cited 2021 Apr 8]. Available from: https://cyps.northyorks.gov.uk/sites/default/files/SEND/Social,%20Emotional%20and%20Mental%20Health/76099_Intervention%20guidanc_proof%2001.pdf

12. Varley D, Wright B, Cooper C, Marshall D, Biggs K, Ali S, et al. Investigating SOcial Competence and Isolation in children with Autism taking part in LEGO-based therapy clubs In School Environments (I-SOCIALISE): study protocol. BMJ Open. 2019 May 1;9(5):e030471.

13. LeGoff DB. Use of LEGO as a therapeutic medium for improving social competence. J Autism Dev Disord. 2004 Oct;34(5):557–71.

14. Owens G, Granader Y, Humphrey A, Baron-Cohen S. LEGO therapy and the social use of language programme: an evaluation of two social skills interventions for children with high functioning autism and Asperger Syndrome. J Autism Dev Disord. 2008 Nov;38(10):1944–57.

15. Ravens-Sieberer U, Wille N, Badia X, Bonsel G, Burström K, Cavrini G, et al. Feasibility, reliability, and validity of the EQ-5D-Y: results from a multinational study. Qual Life Res Int J Qual Life Asp Treat Care Rehabil. 2010 Aug;19(6):887–97.

16. Stevens K. Developing a descriptive system for a new preference-based measure of health-related quality of life for children. Qual Life Res Int J Qual Life Asp Treat Care Rehabil. 2009 Oct;18(8):1105–13.

17. Dolan P. Modeling valuations for EuroQol health states. Med Care. 1997 Nov;35(11):1095–108.

18. Stevens K. Assessing the performance of a new generic measure of health-related quality of life for children and refining it for use in health state valuation. Appl Health Econ Health Policy. 2011 May 1;9(3):157–69.

19. Glick H, Doshi J, Polsky D, Sonnad S. Economic evaluation in clinical trials. second. Oxford: Oxford University Press; 2014.

20. Barrett B, Byford S, Chitsabesan P, Kenning C. Mental health provision for young offenders: service use and cost. Br J Psychiatry J Ment Sci. 2006 Jun;188:541–6.

21. Wright B, Marshall D, Moore DC, Ainsworth H, Hackney L, Adamson J, et al. Autism Spectrum Social Stories In Schools Trial (ASSSIST): study protocol for a feasibility randomised controlled trial analysing clinical and cost-effectiveness of Social Stories in

For peer review only

For peer review only - http://bmjopen.bmj.com/site/about/guidelines.xhtml

mainstream schools. BMJ Open. 2014 Jul 1;4(7):e005952.

22. Marshall D, Wright B, Allgar V, Adamson J, Williams C, Ainsworth H, et al. Social Stories in mainstream schools for children with autism spectrum disorder: a feasibility randomised controlled trial. BMJ Open. 2016 Aug 11;6(8):e011748.

23. NHS Improvement. NHS reference costs 2018/19 [Internet]. [cited 2021 Mar 28]. Available from: https://www.england.nhs.uk/national-cost-collection/

24. Curtis L, Burns A. Unit Costs of Health and Social Care 2018 [Internet]. Canterbury: Univerity of Kent; 2018 [cited 2021 Mar 28]. Available from: https://www.pssru.ac.uk/project-pages/unit-costs/unit-costs-2018/

25. NHS Digital. Prescription Cost Analysis - England 2018 [Internet]. Health and Social Care Information Centre; 2019 [cited 2021 Mar 28]. Available from: https://digital.nhs.uk/data-and-information/publications/statistical/prescription-cost-analysis/2018

26. Family and Childcare Trust. Childcare Survey 2018 [Internet]. Family and Childcare; [cited 2021 Mar 28]. Available from: https://www.familyandchildcaretrust.org/childcare-survey-2018

27. Department for Education. DfE consolidated annual report and accounts 2017 to 2018 [Internet]. London; 2018 [cited 2021 Mar 28]. Available from: https://www.gov.uk/government/publications/dfe-consolidated-annual-report-and-accounts-2017-to-2018

28. National Agreement on Pay and Conditions for Service (Green Book). National Joint council; 2018 p. 225.

29. Employee earnings in the UK (2018) [Internet]. Office for National Statistics; 2019 [cited 2021 Mar 28]. Available from: https://www.ons.gov.uk/employmentandlabourmarket/peopleinwork/earningsandworkinghours/bulletins/annualsurveyofhoursandearnings/2018

30. Rubin DB. Statistical matching using file concatenation with adjusted weights and multiple imputations. J Bus Econ Stat. 1986 Jan 1;4(1):87–94.

31. Manca A, Hawkins N, Sculpher MJ. Estimating mean QALYs in trial-based cost-effectiveness analysis: the importance of controlling for baseline utility. Health Econ. 2005 May;14(5):487–96.

For peer review only - http://bmjopen.bmj.com/site/about/guidelines.xhtml

32. Briggs AH, Wonderling DE, Mooney CZ. Pulling cost-effectiveness analysis up by its bootstraps: A non-parametric approach to confidence interval estimation. Health Econ. 1997;6(4):327–40.

33. Hoch JS, Briggs AH, Willan AR. Something old, something new, something borrowed, something blue: a framework for the marriage of health econometrics and cost-effectiveness analysis. Health Econ. 2002 Jul;11(5):415–30.

34. Fenwick E, O'Brien BJ, Briggs A. Cost-effectiveness acceptability curves--facts, fallacies and frequently asked questions. Health Econ. 2004 May;13(5):405–15.

35. National Institute of Health and Care Excellence (NICE). Guide to the methods of technology appraisal [Internet]. NICE; 2013 [cited 2021 Mar 28]. Available from: https://www.nice.org.uk/process/pmg9/chapter/foreword

36. Costello EJ, Janiszewski S. Who gets treated? Factors associated with referral in children with psychiatric disorders. Acta Psychiatr Scand. 1990 Jun;81(6):523–9.

37. Miller LM, Southam-Gerow MA, Allin RB. Who Stays in Treatment? Child and Family Predictors of Youth Client Retention in a Public Mental Health Agency. Child Youth Care Forum. 2008 Aug 1;37(4):153–70.

38. Sayal K. Annotation: Pathways to care for children with mental health problems. J Child Psychol Psychiatry. 2006 Jul;47(7):649–59.

39. Jongerden L, Simon E, Bodden DHM, Dirksen CD, Bögels SM. Factors associated with the referral of anxious children to mental health care: the influence of family functioning, parenting, parental anxiety and child impairment. Int J Methods Psychiatr Res. 2015 Mar;24(1):46–57.

40. Rinaldi W. Social Use of Language Programme. Infant and primary school teaching pack. Cranleigh: Wendy Rinaldi.; 2004.

41. Strydom A, Bosco A, Vickerstaff V, Hunter R, Poppe M, Ratti V, et al. Clinical and cost effectiveness of staff training in the delivery of Positive Behaviour Support (PBS) for adults with intellectual disabilities, autism spectrum disorder and challenging behaviour - randomised trial. BMC Psychiatry. 2020 Apr 15;20(1):161.

42. LeGoff DB, Sherman M. Long-term outcome of social skills intervention based on interactive LEGO© play. Autism. 2006 Jul 1;10(4):317–29.

For peer review only - http://bmjopen.bmj.com/site/about/guidelines.xhtml

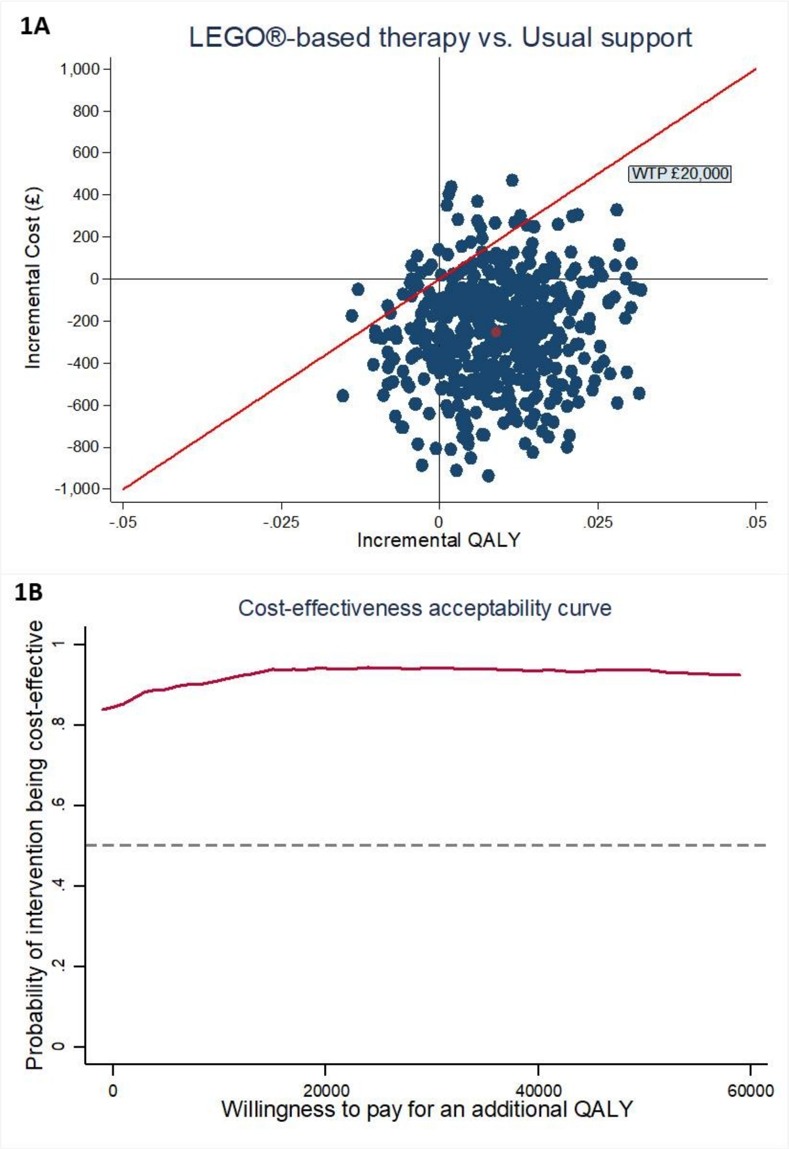

Figure 1: Cost-effectiveness plane and CEAC of primary analysis

71x106mm (300 x 300 DPI)

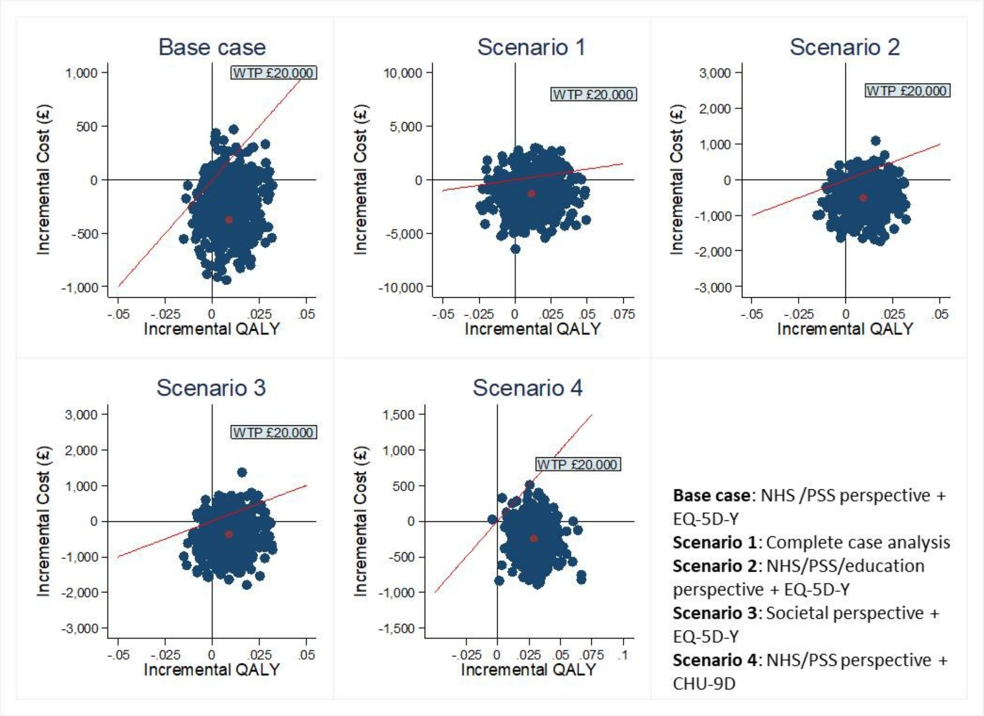

Figure 2: Cost-effectiveness planes of sensitivity analyses

90x70mm (300 x 300 DPI)

Appendix 1: Flow diagram

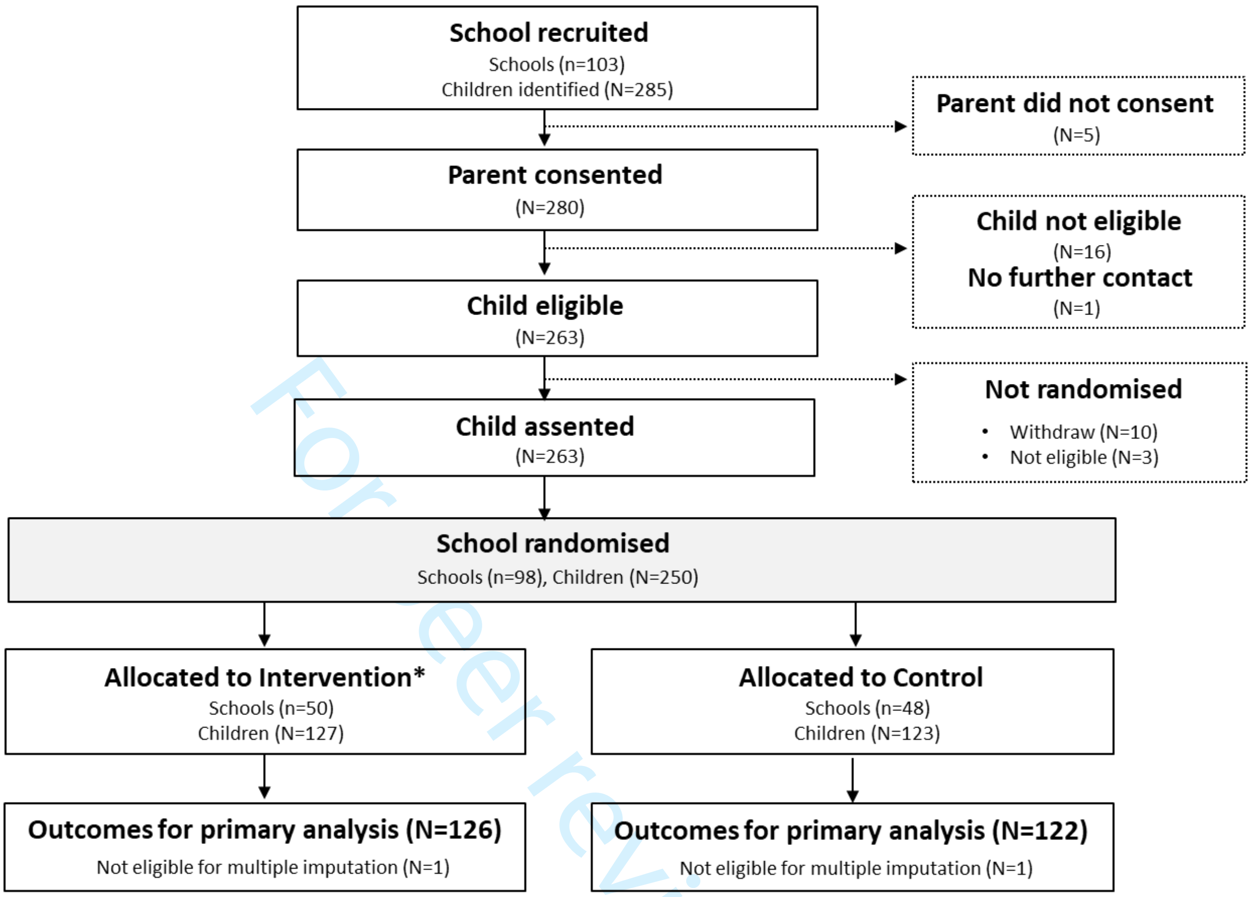

*following ITT principles, school (n=1) children (N-3) allocated to control, but received intervention are included in the control arm

For peer review only

Appendix 2: Intervention costs by trial arm (based on the records without missing data, n=123)

	**Total cost**	**Time per session per CYP (mins) (95% CI)**	**Cost per session per CYP (95% CI)**	**Cost per CYP (95% CI)**
Training costs				
Trainer fee	£4,262		£2.01	
Refreshment costs	£10		£0.00	
Costs for materials and consumables*	£178		£0.08	
Trainer's travel costs	£740		£0.35	
Total	£5,685		£2.45	£25.29 (24.30 – 26.29)
Intervention costs				
LEGO® bricks	£3,903		£1.84	£18.97 (18.23 – 19.70)
Costs for materials and consumable*	£580.5		£0.27	£2.78 (2.68 – 2.89)
Intervention (staff time)				
Planning		3.0 (2.7 – 3.3)	£0.55 (0.50 – 0.60)	£5.46 (4.51 – 6.40)
Set-up		1.7 (1.6 – 1.7)	£0.31 (0.29 – 0.32)	£3.11 (2.76 – 3.46)
Delivery		18.4 (18.1 – 18.6)	£3.53 (3.45 – 3.61)	£35.50 (32.82 – 38.11)
Clear-up		1.8 (1.7 – 1.9)	£0.33 (0.31 - 0.34)	£3.35 (2.98 – 3.71)
Additional work post session**		1.3 (1.1 – 1.5)	£0.26 (0.22 – 0.29)	£2.11 (1.51 – 2.71)
Help from other staff (staff time)				
Set-up and clear-up		0.1 (0.1 – 0.2)	£0.02 (0.01 – 0.03)	£0.25 (0.12 – 0.40)
Additional work post session**		0.1 (0.0 – 0.1)	£0.01 (0.01 – 0.02)	£0.14 (0.04 – 0.23)
Total			£7.12 (6.99 – 7.26)	£71.67 (67.58 – 75.75)

* Costs for pens, paper, file folders, Post-it notes, printing, manual, etc.
** Post-session additional work that was intervention-related, such as administration work, following-up individual CYP, giving brief feedback to teacher, etc.

For peer review only

Appendix 3: Average service use by trial arm (complete case, n=139)

	Unit	Baseline		0-20 weeks		20-52 weeks	
		LEGO® based therapy, N=80 Mean (sd)	Usual care, N=59 Mean (sd)	LEGO® based therapy. N=80 Mean (sd)	Usual support N=59 Mean (sd)	LEGO® based therapy, N=80 Mean (sd)	Usual support N=59 Mean (sd)
**NHS and PSS**							
Community-based services							
CAMHS related	Session	0.70 (2.76)	0.31 (0.88)	0.23 (0.88)	0.36 (1.06)	0.63 (2.81)	0.54 (2.46)
Non-CAMHS related							
GP	Appointment	0.45 (0.97)	0.24 (0.50)	0.39 (1.02)	0.22 (0.49)	0.71 (1.41)	0.66 (0.96)
Allied health professionals	Appointment	0.44 (0.93)	0.68 (2.20)	0.43 (1.00)	0.34 (0.71)	0.78 (1.96)	0.69 (1.56)
Social care services	Appointment	0.49 (1.65)	0.47 (1.34)	0.26 (1.00)	0.39 (2.11)	0.99 (2.62)	0.80 (2.06)
Hospital-based services / acute services							
Emergency services	Visit	0.19 (0.80)	0.12 (0.46)	0.13 (0.43)	0.03 (0.26)	0.20 (0.60)	0.17 (0.62)
Inpatient stay							
Mental health related	Night	-	-	-	-	-	-
Non-mental health related	Night	0.01 (0.11)	-	0.01 (0.11)	-	-	-
Outpatient visit / day case							
Mental health related	Visit	0.06 (0.37)	0.10 (0.44)	0.08 (0.38)	0.07 (0.31)	0.05 (0.22)	0.29 (1.37)
Non-mental health related	Visit	0.31 (1.71)	0.17 (0.59)	0.19 (0.75)	0.24 (0.63)	0.09 (0.40)	0.24 (0.95)
Medication							
Mental health related	Type	0.36 (0.80)	0.20 (0.48)	0.43 (0.81)	0.27 (0.55)	0.48 (0.87)	0.32 (0.51)
Non-mental health related	Type	0.33 (0.62)	0.41 (0.93)	0.41 (0.90)	0.53 (1.33)	0.38 (0.79)	0.59 (1.23)
**Education system related**							

For peer review only - http://bmjopen.bmj.com/site/about/guidelines.xhtml

School-based health	Hour	2.60 (10.00)	2.17 (6.94)	1.40 (5.80)	3.12 (10.51)	8.80 (32.99)	2.17 (10.06)
General support*	Hour	8.54 (7.26)	7.93 (12.68)	7.48 (9.25)	9.37 (17.34)	18.94 (30.32)	27.32 (45.13)
Intervention support*	Hour	5.50 (10.50)	4.66 (6.74)	6.78 (8.28)	7.15 (11.03)	8.37 (7.97)	7.72 (10.56)
**Private expanses – out of pocket**							
Childcare	Session	3.05 (7.35)	4.10 (11.05)	4.14 (9.30)	5.98 (12.37)	5.86 (13.58)	12.29 (25.20)
**Productivity**							
Parental productivity	Day	0.45 (1.18)	0.31 (0.93)	0.45 (1.04)	0.52 (1.51)	0.58 (1.13)	0.59 (1.40)

CAMHS: Child and Adolescent Mental Health Services, including child psychiatrist, child psychotherapist, child psychologist, clinical psychologist, mental health nurse, family therapist, and Primary mental health worker (PMHW)

Allied health professionals included community nurse, community paediatrician, occupational therapist, physiotherapist, and Speech and Language therapist

Social care services included social care worker, home care worker, family support worker, drug and alcohol support worker, and Helpline (e.g. Samaritans)

Childcare included paid childcare, after school club, religious club, and special clubs for autism children

*based on 117 teacher-reported questionnaires (68 from I-socialise arm and 48 from usual care arm)

For peer review only

Appendix 4: EQ-5D-Y responses by trial arms by data collection time points

Usual support (n=59)	Baseline			20 Weeks			52 Weeks		
	Level 1 n (%)	Level 2 n (%)	Level 3 n (%)	Level 1 n (%)	Level 2 n (%)	Level 3 n (%)	Level 1 n (%)	Level 2 n (%)	Level 3 n (%)
Mobility	52 (88.1)	7 (11.9)	-	46 (78.0)	13 (22.0)	-	46 (78.0)	13 (22.0)	-
Self-care	17 (28.8)	32 (54.2)	10 (17.0)	16 (27.1)	37 (62.7)	6 (10.2)	14 (23.7)	35 (59.3)	10 (17.0)
Usual activity	25 (42.4)	29 (49.1)	5 (8.5)	25 (42.4)	27 (45.8)	7 (11.8)	22 (37.3)	30 (50.8)	7 (11.9)
Pain/discomfort	45 (76.3)	13 (22.0)	1 (1.7)	37 (62.7)	22 (37.3)	-	38 (64.4)	21 (35.6)	-
Anxiety/depression	15 (25.4)	35 (59.3)	9 (15.3)	16 (27.1)	34 (57.6)	9 (15.3)	18 (30.5)	26 (44.1)	15 (25.4)
**LEGO® based therapy (n=80)**	**Baseline**			**20 Weeks**			**52 Weeks**		
	Level 1 n (%)	Level 2 n (%)	Level 3 n (%)	Level 1 n (%)	Level 2 n (%)	Level 3 n (%)	Level 1 n (%)	Level 2 n (%)	Level 3 n (%)
Mobility	66 (82.5)	11 (13.7)	3 (3.8)	61 (76.2)	16 (20.0)	3 (3.8)	64 (80.0)	13 (16.2)	3 (3.8)
Self-care	25 (31.3)	36 (45.0)	19 (23.7)	26 (32.5)	42 (52.5)	12 (15.0)	29 (36.3)	36 (45.0)	15 (18.7)
Usual activity	33 (41.2)	41 (51.2)	6 (7.5)	35 (43.7)	36 (45.0)	9 (11.3)	43 (53.7)	33 (41.3)	4 (5.0)
Pain/discomfort	64 (80.0)	14 (17.5)	2 (2.5)	63 (78.7)	13 (16.3)	4 (5.0)	59 (73.8)	19 (23.7)	2 (2.5)
Anxiety/depression	40 (50.0)	33 (41.3)	7 (8.7)	37 (46.2)	34 (42.5)	9 (11.3)	39 (48.7)	29 (36.3)	12 (15.0)

*Level 1: none, Level 2: some, Level 3: extreme

For peer review only - http://bmjopen.bmj.com/site/about/guidelines.xhtml

Appendix 5: CHU-9D responses by trial arms by data collection time points

Usual support (n=45)	Baseline					20 weeks					52 weeks				
	Level 1	Level 2	Level 3	Level 4	Level 5	Level 1	Level 2	Level 3	Level 4	Level 5	Level 1	Level 2	Level 3	Level 4	Level 5
Worried	20 (44.4)	16 (35.6)	7 (15.6)	-	2 (4.4)	22 (48.9)	12 (26.7)	5 (11.1)	3 (6.7)	3 (6.7)	26 (57.8)	13 (28.9)	1 (2.2)	3 (6.7)	2 (4.4)
Sad	32 (71.1)	8 (17.8)	2 (4.4)	2 (4.4)	1 (2.2)	29 (64.4)	6 (13.3)	5 (11.1)	4 (8.9)	1 (2.2)	34 (75.6)	4 (8.9)	2 (4.4)	1 (2.2)	4 (8.9)
Annoyed	27 (60.0)	13 (28.9)	4 (8.9)	-	1 (2.2)	27 (60.0)	10 (22.2)	5 (11.1)	-	3 (6.7)	27 (60.0)	10 (22.2)	5 (11.1)	2 (4.4)	1 (2.2)
Tired	9 (20.0)	15 (33.3)	8 (17.8)	5 (11.1)	8 (17.8)	12 (26.7)	13 (28.9)	9 (20.0)	5 (11.1)	6 (13.3)	10 (22.2)	16 (35.6)	6 (13.3)	6 (13.3)	7 (15.6)
Pain	28 (62.2)	6 (13.3)	6 (13.3)	3 (6.7)	2 (4.4)	21 (46.7)	11 (24.4)	4 (8.9)	2 (4.4)	7 (15.6)	24 (53.3)	12 (26.7)	7 (15.6)	-	2 (4.4)
Sleep	21 (46.7)	12 (26.7)	5 (11.1)	3 (6.7)	4 (8.9)	20 (44.4)	3 (6.7)	7 (15.6)	9 (20.0)	6 (13.3)	17 (37.8)	16 (35.6)	4 (8.9)	4 (8.9)	4 (8.9)
Daily routine	23 (51.1)	12 (26.7)	4 (8.9)	3 (6.7)	3 (6.7)	19 (42.2)	11 (24.4)	7 (15.6)	4 (8.9)	4 (8.9)	24 (53.3)	10 (22.2)	5 (11.1)	3 (6.7)	3 (6.7)
Work	27 (60.0)	15 (33.3)	2 (4.4)	1 (2.2)	-	23 (51.1)	11 (24.4)	7 (15.6)	1 (2.2)	3 (6.7)	28 (62.2)	7 (15.6)	7 (15.6)	1 (2.2)	2 (4.4)
Able to join activities	23 (51.1)	6 (13.3)	10 (22.2)	3 (6.7)	3 (6.7)	10 (22.2)	14 (31.1)	10 (22.2)	9 (20.0)	2 (4.4)	20 (44.4)	10 (22.2)	10 (22.2)	1 (2.2)	4 (8.9)
**LEGO® based therapy**	**Baseline**					**20 weeks**					**52 weeks**				
**(n=51)**	**Level 1**	**Level 2**	**Level 3**	**Level 4**	**Level 5**	**Level 1**	**Level 2**	**Level 3**	**Level 4**	**Level 5**	**Level 1**	**Level 2**	**Level 3**	**Level 4**	**Level 5**
Worried	34 (66.7)	8 (15.7)	3 (5.9)	2 (3.9)	4 (7.8)	33 (64.7)	6 (11.8)	7 (13.7)	1 (2.0)	4 (7.8)	34 (66.7)	7 (13.7)	6 (11.8)	3 (5.9)	1 (2.0)
Sad	40 (78.4)	5 (9.8)	2 (3.9)	1 (2.0)	3 (5.9)	37 (72.5)	5 (9.8)	5 (9.8)	1 (2.0)	3 (5.9)	36 (70.6)	6 (11.8)	6 (11.8)	2 (3.9)	1 (2.0)
Annoyed	37 (72.5)	7 (13.7)	4 (7.8)	2 (3.9)	1 (2.0)	34 (66.7)	10 (19.6)	5 (9.8)	-	2 (3.9)	37 (72.5)	8 (15.7)	3 (5.9)	1 (2.0)	2 (3.9)
Tired	14 (27.5)	19 (37.3)	5 (9.8)	3 (5.9)	10 (19.6)	17 (33.3)	19 (37.3)	4 (7.8)	7 (13.7)	4 (7.8)	13 (25.5)	19 (37.3)	6 (11.8)	4 (7.8)	9 (17.6)
Pain	33 (64.7)	9 (17.6)	3 (5.9)	1 (2.0)	5 (9.8)	28 (54.9)	9 (17.6)	7 (13.7)	3 (5.9)	4 (7.8)	27 (52.9)	13 (25.5)	6 (11.8)	3 (5.9)	2 (3.9)
Sleep	26 (51.0)	10 (19.6)	6 (11.8)	3 (5.9)	6 (11.8)	23 (45.1)	8 (15.7)	8 (15.7)	4 (7.8)	8 (15.7)	20 (39.2)	11 (21.6)	11 (21.6)	3 (5.9)	6 (11.8)
Daily routine	29 (56.9)	8 (15.7)	4 (7.8)	4 (7.8)	6 (11.8)	24 (47.1)	15 (29.4)	7 (13.7)	3 (5.9)	2 (3.9)	27 (52.9)	12 (23.5)	8 (15.7)	3 (5.9)	1 (2.0)
Work	34 (66.7)	7 (13.7)	6 (11.8)	1 (2.0)	3 (5.9)	33 (64.7)	10 (19.6)	5 (9.8)	3 (5.9)	-	32 (62.7)	11 (21.6)	4 (7.8)	2 (3.9)	2 (3.9)
Able to join activities	30 (58.8)	6 (11.8)	5 (9.8)	4 (7.8)	6 (11.8)	24 (47.1)	8 (15.7)	6 (11.8)	8 (15.7)	5 (9.8)	20 (39.2)	10 (19.6)	7 (13.7)	8 (15.7)	6 (11.8)

* Level 1: No, Level 2: A little bit, Level 3: A bit, Level 4: Quite a lot, Level 5 Very

For peer review only - http://bmjopen.bmj.com/site/about/guidelines.xhtml

Appendix 6: Sensitivity analyses

LEGO® based therapy vs. usual support	Incremental costs (£), (95% CI)	Incremental QALYs (95% CI)	ICER (£/QALY gained), (95% CI)
Scenario 1: Complete case analysis from NHS perspective	-1,280<br>(-4,578 to 2,081)	0.011<br>(-0.017 to 0.040)	Dominant
Scenario 2: CUA from NHS and education perspective	-511<br>(-1,452 to 392)	0.009<br>(-0.008 to 0.028)	Dominant
Scenario 3: CUA from societal perspective	-376<br>(-1,377 to 595)	0.009<br>(-0.008, 0.028)	Dominant
Scenario 4: Assume outcomes were measured using CHU-9D	-246<br>(-719 to 246)	0.029<br>(0.009 to 0.049)	Dominant

For peer review only

For peer review only - http://bmjopen.bmj.com/site/about/guidelines.xhtml

CHEERS Checklist

Items to include when reporting economic evaluations of health interventions

The ISPOR CHEERS Task Force Report, Consolidated Health Economic Evaluation Reporting Standards (CHEERS)—Explanation and Elaboration: A Report of the ISPOR Health Economic Evaluations Publication Guidelines Good Reporting Practices Task Force, provides examples and further discussion of the 24-item CHEERS Checklist and the CHEERS Statement. It may be accessed via the Value in Health or via the ISPOR Health Economic Evaluation Publication Guidelines – CHEERS: Good Reporting Practices webpage: http://www.ispor.org/TaskForces/EconomicPubGuidelines.asp

**Section/item**	**Item No**	**Recommendation**	**Reported on page No/ line No**
**Title and abstract**			
Title	1	Identify the study as an economic evaluation or use more specific terms such as "cost-effectiveness analysis", and describe the interventions compared.	Page 1
Abstract	2	Provide a structured summary of objectives, perspective, setting, methods (including study design and inputs), results (including base case and uncertainty analyses), and conclusions.	Page 2
**Introduction**			
Background and objectives	3	Provide an explicit statement of the broader context for the study. Present the study question and its relevance for health policy or practice decisions.	Page 4, Section 1
**Methods**			
Target population and subgroups	4	Describe characteristics of the base case population and subgroups analysed, including why they were chosen.	Page 4, Section 2.1
Setting and location	5	State relevant aspects of the system(s) in which the decision(s) need(s) to be made.	Page 5, Section 2.1
Study perspective	6	Describe the perspective of the study and relate this to the costs being evaluated.	Page 6, Section 2.4
Comparators	7	Describe the interventions or strategies being compared and state why they were chosen.	Page 5, Section 2.1
Time horizon	8	State the time horizon(s) over which costs and consequences are being evaluated and say why	Page 7, Section 2.4

For peer review only

		appropriate.	
Discount rate	9	Report the choice of discount rate(s) used for costs and outcomes and say why appropriate.	Page 7, Section 2.4
Choice of health outcomes	10	Describe what outcomes were used as the measure(s) of benefit in the evaluation and their relevance for the type of analysis performed.	Sections 2.3 and 2.4
Measurement of effectiveness	11a	Single study-based estimates: Describe fully the design features of the single effectiveness study and why the single study was a sufficient source of clinical effectiveness data.	Page 4, Section 2.1
	11b	Synthesis-based estimates: Describe fully the methods used for identification of included studies and synthesis of clinical effectiveness data.	NA
Measurement and valuation of preference based outcomes	12	If applicable, describe the population and methods used to elicit preferences for outcomes.	Page5, Section 2.3
Estimating resources and costs	13a	*Single study-based economic evaluation*: Describe approaches used to estimate resource use associated with the alternative interventions. Describe primary or secondary research methods for valuing each resource item in terms of its unit cost. Describe any adjustments made to approximate to opportunity costs.	NA
	13b	*Model-based economic evaluation*: Describe approaches and data sources used to estimate resource use associated with model health states. Describe primary or secondary research methods for valuing each resource item in terms of its unit cost. Describe any adjustments made to approximate to opportunity costs.	Page7, Section 2.4
Currency, price date, and conversion	14	Report the dates of the estimated resource quantities and unit costs. Describe methods for adjusting estimated unit costs to the year of reported costs if necessary. Describe methods for converting costs into a common currency base and the exchange rate.	NA
Choice of model	15	Describe and give reasons for the specific type of decision-analytical model used. Providing a figure to show model structure is strongly recommended.	NA

For peer review only

Assumptions	16	Describe all structural or other assumptions underpinning the decision-analytical model.	NA
Analytical methods	17	Describe all analytical methods supporting the evaluation. This could include methods for dealing with skewed, missing, or censored data; extrapolation methods; methods for pooling data; approaches to validate or make adjustments (such as half cycle corrections) to a model; and methods for handling population heterogeneity and uncertainty.	Sections 2.5 and 2.6
Results			
Study parameters	18	Report the values, ranges, references, and, if used, probability distributions for all parameters. Report reasons or sources for distributions used to represent uncertainty where appropriate. Providing a table to show the input values is strongly recommended.	NA
Incremental costs and outcomes	19	For each intervention, report mean values for the main categories of estimated costs and outcomes of interest, as well as mean differences between the comparator groups. If applicable, report incremental cost-effectiveness ratios.	Sections 3.2, 3.3 and 3.4
Characterising uncertainty	20a	*Single study-based economic evaluation*: Describe the effects of sampling uncertainty for the estimated incremental cost and incremental effectiveness parameters, together with the impact of methodological assumptions (such as discount rate, study perspective).	Section 3.4 and 3.5
	20b	*Model-based economic evaluation*: Describe the effects on the results of uncertainty for all input parameters, and uncertainty related to the structure of the model and assumptions.	NA
Characterising heterogeneity	21	If applicable, report differences in costs, outcomes, or cost- effectiveness that can be explained by variations between subgroups of patients with different baseline characteristics or other observed variability in effects that are not reducible by more information.	NA
**Discussion**			
Study findings, limitations,	22	Summarise key study findings and describe how they support the conclusions reached. Discuss limitations	Page 12, Section 4

For peer review only

generalisability, and current knowledge		and the generalisability of the findings and how the findings fit with current knowledge.	
**Other**			
Source of funding	23	Describe how the study was funded and the role of the funder in the identification, design, conduct, and reporting of the analysis. Describe other non-monetary sources of support.	Page 16
Conflicts of interest	24	Describe any potential for conflict of interest of study contributors in accordance with journal policy. In the absence of a journal policy, we recommend authors comply with International Committee of Medical Journal Editors recommendations.	Page 16

For consistency, the CHEERS Statement checklist format is based on the format of the CONSORT statement checklist

The ISPOR CHEERS Task Force Report provides examples and further discussion of the 24-item CHEERS Checklist and the CHEERS Statement. It may be accessed via the Value in Health link or via the ISPOR Health Economic Evaluation Publication Guidelines – CHEERS: Good Reporting Practices webpage: http://www.ispor.org/TaskForces/EconomicPubGuidelines.asp

The citation for the CHEERS Task Force Report is:
Husereau D, Drummond M, Petrou S, et al. Consolidated health economic evaluation reporting standards (CHEERS)—Explanation and elaboration: A report of the ISPOR health economic evaluations publication guidelines good reporting practices task force. Value Health 2013;16:231-50.

For peer review only

BMJ Open

Cost-utility analysis of LEGO® based therapy for school children and young people with autism spectrum disorder: results from a randomised controlled trial

Journal:	*BMJ Open*
Manuscript ID	bmjopen-2021-056347.R1
Article Type:	Original research
Date Submitted by the Author:	22-Nov-2021
Complete List of Authors:	Wang, Han-I; University of York, Department of Health Sciences<br>Wright, Barry; University of York; Leeds and York Partnership NHS Foundation Trust<br>Bursnall, Matthew; The University of Sheffield, School of Health and Related Research (ScHARR)<br>Cooper, Cindy; The University of Sheffield<br>Kingsley, Ellen; Leeds and York Partnership NHS Foundation Trust, COMIC Research Team<br>Le Couteur, Ann; Newcastle University,<br>Teare, Dawn; Newcastle University Faculty of Medical Sciences<br>Biggs, Katie; The University of Sheffield, Clinical Trials Research Unit, ScHARR<br>McKendrick, Kirsty; The University of Sheffield, Clinical Trials Unit<br>Gomez de la Cuesta, Gina; Play Included CIC<br>Chater, Tim; University of Liverpool<br>Barr, Amy; The University of Sheffield<br>Solaiman, Kiera; The University of Sheffield<br>Packham, Anna; The University of Sheffield<br>Marshall, David; University of York, Lime Trees CAMHS, Research Team<br>Varley, Danielle; University of York Department of Health Sciences<br>Nekooi, Roshanak; Leeds and York Partnership NHS Foundation Trust<br>Gilbody, Simon; University of York, Department of Health Sciences<br>Parrott, Steve; University of York
**Primary Subject Heading**:	Health economics
Secondary Subject Heading:	Mental health
Keywords:	HEALTH ECONOMICS, Child & adolescent psychiatry < PSYCHIATRY, Clinical trials < THERAPEUTICS

SCHOLARONE™
Manuscripts

I, the Submitting Author has the right to grant and does grant on behalf of all authors of the Work (as defined in the below author licence), an exclusive licence and/or a non-exclusive licence for contributions from authors who are: i) UK Crown employees; ii) where BMJ has agreed a CC-BY licence shall apply, and/or iii) in accordance with the terms applicable for US Federal Government officers or employees acting as part of their official duties; on a worldwide, perpetual, irrevocable, royalty-free basis to BMJ Publishing Group Ltd ("BMJ") its licensees and where the relevant Journal is co-owned by BMJ to the co-owners of the Journal, to publish the Work in this journal and any other BMJ products and to exploit all rights, as set out in our licence.

The Submitting Author accepts and understands that any supply made under these terms is made by BMJ to the Submitting Author unless you are acting as an employee on behalf of your employer or a postgraduate student of an affiliated institution which is paying any applicable article publishing charge ("APC") for Open Access articles. Where the Submitting Author wishes to make the Work available on an Open Access basis (and intends to pay the relevant APC), the terms of reuse of such Open Access shall be governed by a Creative Commons licence – details of these licences and which Creative Commons licence will apply to this Work are set out in our licence referred to above.

Other than as permitted in any relevant BMJ Author's Self Archiving Policies, I confirm this Work has not been accepted for publication elsewhere, is not being considered for publication elsewhere and does not duplicate material already published. I confirm all authors consent to publication of this Work and authorise the granting of this licence.

For peer review only

Cost-utility analysis of LEGO® based therapy for school children and young people with autism spectrum disorder: results from a randomised controlled trial

Authors

Han-I Wang[1], Barry Wright [1,2], Matthew Bursnall[3], Cindy Cooper[3], Ellen Kingsley[2], Ann Le Couteur[4], Dawn Teare[3], Katie Biggs[3], Kirsty McKendrick[3], Gina Gomez de la Cuesta[5], Tim Chater[3], Amy Barr[3], Kiera Solaiman[3], Anna Packham[3], David Marshall[1], Danielle Varley[1], Roshanak Nekooi[2], Simon Gilbody[1], Steve Parrott[1]

[1]University of York, York, UK

[2]Leeds and York Partnership NHS Foundation Trust, Leeds, UK

[3]CTRU, University of Sheffield, Sheffield, UK

[4]Newcastle University, Newcastle, UK

[5]Play Included CIC

Corresponding author

Name: Dr. Han-I Wang

Email: han-i.wang@york.ac.uk

Address: Department of Health Sciences, Seebohm Rowntree Building, University of York, Heslington, York, YO10 5DD

Tel: 01904-321817

Key words: cost, effectiveness, EQ-5D-Y, LEGO, Play Brick Therapy, autism, ASD, RCT

Word count: 4472

For peer review only - http://bmjopen.bmj.com/site/about/guidelines.xhtml

Abstract

Objectives: To assess the cost-effectiveness of LEGO® based therapy compared to usual support.

Design: cost-utility analysis alongside randomised control trial.

Setting: mainstream primary and secondary schools in the UK.

Participants: 248 children and young people (CYP) with autism spectrum disorder (ASD) aged 7-15 years.

Intervention: LEGO® based therapy is a group social skills intervention designed specifically for CYP with ASD. Through play, CYP learn to use the skills such as joint attention, sharing, communication, and group problem solving. CYP randomised to the intervention arm received 12 weekly sessions of LEGO® based therapy and usual support, while CYP allocated to control arm received usual support only.

Main outcome measures: Average costs based on NHS and personal social services perspective and quality adjusted life years (QALYs) measured by EQ-5D-Y over time horizon of one year were collected during the trial. Incremental cost-effectiveness ratio (ICER) was calculated, and non-parametric bootstrapping was conducted. The uncertainty around the ICER estimates was presented using cost-effectiveness acceptability curve (CEAC). A set of sensitivity analyses were conducted to assess the robustness of the primary findings.

Results: After adjustment and bootstrapping, on average, CYP in LEGO® based therapy group incurred less costs (incremental cost was -£251 (95% CI -£752 to £268)) and gained marginally improvement in QALYs (QALYs gained 0.009 (95% CI −0.008 to 0.028)). The CEAC shows that the probability of LEGO® based therapy being cost-effective was 94% at the WTP threshold of £20,000 per QALY gained. Results of sensitivity analyses were consistent with the primary outcomes.

Conclusion: Compared to usual support, LEGO® based therapy produced marginal reduction in costs and improvement in QALYs. Results from both primary and sensitivity analyses suggested that LEGO® based therapy was likely to be cost-effective.

Trial registration: ISRCTN64852382

For peer review only - http://bmjopen.bmj.com/site/about/guidelines.xhtml

Strengths and limitations of this study

1. The first economic evaluation study of LEGO® based therapy.
2. The data are from a large sample size trial in ASD, and the method followed published best-practice guidance.
3. The study accounts for the costs measured from a range of perspectives and the QALYs measured by different instruments.
4. Resource use data were collected retrospectively and may be affected by inaccurate recall.
5. Further model-based evaluation is required to assess long term cost-effectiveness of LEGO® based therapy.

For peer review only

For peer review only - http://bmjopen.bmj.com/site/about/guidelines.xhtml

1. Background

Autism spectrum disorders (ASDs) are a group of lifelong developmental conditions defined by impairments in social interactions, communication skills, and presence of restricted and stereotypical behaviours (1). It is estimated that around 120,000 children and young people (CYP)(1.1% of total CYP) in the UK have a diagnosis of ASD (2–4). CYP with ASD have differing health and quality of life outcomes to neurotypically developing people and over their lifetime this is likely to have a financial impact on their families or carers. In the UK, the annual cost of supporting children with ASD has been estimated at between £3.1 and £3.4 billion (in 2011 value) with higher values when there is associated intellectual disability. The main cost driver of the annual support cost was special education (47%) followed by parental productivity loss (12%). Medical services accounted for only 4 % of the total (5).

Due to the substantial financial burden borne by the health care system, education system, and families of CYP with ASD, the Lancet Psychiatry Commission (LPC) emphasizes the need to not only focus on the effectiveness of mental health services, but also on their economic benefits (6). However, with the evolving intervention landscape of ASD, only two economic evaluation studies were found for CYP with ASD (7). One investigated the cost-effectiveness of a communication intervention (8) and another investigated an early intervention programme (9). These preliminary studies suggested that early intervention for children with suspected ASD was cost-effective, but communication-focused therapy for preschool children with ASD was not, with more research being recommended.

LEGO® based therapy (10) is a group based social skills intervention specifically designed for CYP with ASD which does not rely on adult-led teaching of skills. It has become very popular in the UK with many local authorities now recommending its use in schools (11). Despite the growing interest and substantial economic burden of ASD, no economic evaluation study has been done for LEGO® based therapy. Hence, the aim of this study was to access the cost-effectiveness of LEGO® based therapy. This paper reports the economic evaluation results of LEGO® based therapy for CYP with ASD alongside the Investigating SOcial Competence and Isolation in children with Autism taking part in LEGO® based therapy clubs In School Environments (I-SOCIALISE) trial (12).

2. Methods

2.1 Trial design and participants

This economic evaluation was embedded in the I-SOCIALISE trial, a multisite, pragmatic, two-arm, school-level cluster randomised controlled trial (RCT) for CYP with ASD. Details of

For peer review only - http://bmjopen.bmj.com/site/about/guidelines.xhtml

I-SOCIALISE trial have been published elsewhere (12). In short, CYP between the ages of seven and 15 years with a diagnosis of ASD were recruited from mainstream primary and secondary schools in the North of England between October 2018 and May 2019. Parents/guardians and schools were invited to speak on the phone or face-to-face to discuss the eligibility of CYP in their school and their potential involvement in the study. CYP were included in the study if they met study inclusion criteria which included aged between seven and 15 years with a clinical diagnosis of ASD, a score of 15 or higher on the Social Communication Questionnaire (SCQ), the ability to understand simple instructions, no serious impairments which would prevent participation, and were attending mainstream schools in the north of England (see Appendix 1). CYP in schools that were allocated by remote randomisation to the intervention arm received a one-hour session of LEGO® based therapy in school once per week for the 12-week period. On average, around three CYP were in each session. The decision about number of sessions and the duration per session were based on recommendations of the co-author and experienced LEGO-therapy trainer (Gina Comez de la Cuesta), the published traning manual and in line with previous studies on school based intervention (such as Scocial Stories (13)). There was some flexibility in weekly delivery to accommodate school timetables. CYP also received usual support, while CYP in schools allocated to the control arm received usual support only. Usual support includes any support the CYP with ASD was receiving at the time from school, general practitioners (GPs) or other professionals. The usual support from school includes the support from specialist teaching teams for autism as well as other interventions such as the Picture Exchange Communication System, visual supports and timetables and Social Stories. To investigate the efficacy and cost-effectiveness of LEGO® based therapy whilst controlling for any impact obtained from usual support, CYP received LEGO® based therapy and usual support were compared to usual support alone, rather than to another similar intervention. Informed consent and baseline measurements were obtained and completed prior to school randomisation. All CYP were followed up 20 and 52 weeks after randomisation and completed further outcome measures. A flowchart of the study can be found in Appendix 2.

2.2 Intervention

LEGO® based therapy is a group social skills intervention designed specifically for CYP with social communication difficulties such as ASD. CYP build LEGO® models together in small groups with light facilitation by a trained adult (14,15). The CYP work together taking on one of three roles: the engineer, who reads the LEGO® set instructions; the supplier, who finds the correct pieces according to the instructions from the engineer; and the builder, who builds the model with the pieces from the supplier and the instructions from the engineer. Key elements of the intervention include the use of CYP-led collaborative building and learning through play which promote the learning and use of such skills as joint attention,

For peer review only - http://bmjopen.bmj.com/site/about/guidelines.xhtml

sharing, communication, and group problem solving. The trained adult takes a guiding role rather than an explicitly directive one, allowing the CYP to work together and solve their own challenges. Group rules and rewards are used to foster motivation and engagement in social interactions.

2.3 Outcomes

The health outcomes for the current study were quality adjusted life years (QALYs) measured by the EQ-5D-Y (3L proxy version) (16) and the Child Health Utility 9D (CHU-9D) (17). EQ-5D-Y (3L proxy version) is a five-item with three-severity level questionnaire that allows a proxy person (i.e. parent/guardian) to complete the measure for CYP. The EQ-5D-Y instrument comprises five dimensions (mobility, looking after themselves, doing usual activities, having any pain or discomfort, and feeling worried or sad) and has been shown to be a reliable and valid instrument for use in CYP and adolescents (16). The CHU-9D is a CYP-completed nine-item questionnaire comprising nine dimensions: worried, sad, pain, tired, annoyed, schoolwork/homework, sleep, daily routine, and able to join in activities (17).

Individual-level responses to EQ-5D-Y and CHU-9D were used to estimate utilities based on UK population valuation sets (18,19). A utility represents a CYP's health state and is constrained between 0 and 1, where 0 refers to death and 1 perfect health. The estimated utilities at baseline and follow-up were further joint using the area under the curve approach (20) to calculate QALYs.

2.4 cost measurement

Two cost perspectives were considered in this study: 1) a NHS and personal social service (NHS/PSS) perspective, which included costs related to healthcare (including hospital-based services, such as inpatient stays, outpatient visits and emergency care, and services outside a hospital setting, such as GP visits, Child and Adolescent Mental Health Services, and services provided by allied health professionals (e.g. community paediatrician)) and social services (including social care worker, home care worker, family support worker, and Helpline (e.g. Samaritans)), and 2) a societal perspective, which additionally included costs of education-related services, parental out-of pocket expenses (such as childcare and private courses), and parental productivity costs (time off work due to child's autism) without taking social charges (any payments or contributions for social benefits) into consideration..

Cost of the intervention

Cost of the intervention included the cost of training and the cost of delivering LEGO® based

For peer review only - http://bmjopen.bmj.com/site/about/guidelines.xhtml

therapy. Training costs were measured by the time spent by the trainer and included travel costs and the cost of materials used in the training. Costs associated with delivering the LEGO® based therapy were measured based on the time spent by facilitators to plan and conduct sessions and included relevant overhead costs. All relevant data were collected using the tailored questionnaires completed by the study team and therapists.

Cost of the service use

Service use was collected using the tailored questionnaires (completed by the parent/guardian and separately by an associated teacher of each CYP in the study who knew the CYP well), which was originally developed based on Barrett's study (21) and has been successfully adapted for use in school-based trials (22,23). The parent/guardian-completed questionnaire captured data on the use of health and social services, school-based services (including school-based health services, general and intervention support), parental private expenses, and parental productivity costs. Teacher-completed questionnaires captured any school-based interventions/support and the implications of a CYP's behaviour on school resource.

Service use was multiplied by unit costs to arrive at total cost in each arm. Unit costs of health and social service use were obtained from published sources (i.e. Reference Cost 2018 (24), Personal Social Services Research Unit 2018 (25), and Prescription Cost Analysis – England 2018 (26)), national survey (i.e. Childcare Costs Survey 2018 (27)), and government departments (i.e. Department for Education 2018 (28) and Green Book 2018 (29)). Privately paid services were separately estimated via market prices, while productivity losses were calculated using the human capital approach, which involves multiplying time off work by UK average salary (30).

All the costs were expressed in 2018 UK sterling. Discounting of costs and QALYs was not applied, as the study time horizon was one year.

2.5 Missing data

All eligible CYPs who had both utility and cost data at any time point were referred to as complete cases. While, the eligible CYPs who had missing utility and cost data but had complete baseline assessments were referred to as base case. The identified missing utility and cost data were imputed using multiple imputation method via chained equations (31). Imputation was based on trial arm, age, gender, study site, and utility scores and Social Communication Questionnaire (SCQ) scores at baseline.

For peer review only - http://bmjopen.bmj.com/site/about/guidelines.xhtml

For peer review only

2.6 Statistical and economic analyses

The primary analysis was to calculate incremental cost-effectiveness ratio (ICER) based on the costs from NHS/PSS perspective and the QALYs measured by EQ-5D-Y. To account for uncertainty around ICER and imbalanced utility and costs at baseline, seemingly unrelated regression equations (SURE) that adjusted standard errors for clustering and controlled for baseline utility (32), costs, age, gender, and SCQ scores were bootstrapped 5,000 times. The SURE approach was recommended by Glick and colleagues (19), which considers the distribution of the dependent variable and any correlation found between cost and QALY outcomes. While non-parametric bootstrap re-sampling method was suggested by Briggs and colleagues (33), as the distribution of regression residuals was likely to be skewed (34). The 5,000 bootstrapped iterations were represented graphically on the cost-effectiveness plane (CE-plane), and the cost-effectiveness acceptability curve (CEAC) was generated by plotting the probability of the intervention being cost-effective over a range of willingness-to-pay (WTP) thresholds (35). The calculated ICERs were against the national WTP threshold of £20,000-£30,000 per QALY gained to decide whether the LEGO® based therapy is cost-effectiveness (36).

To assess the robustness of our findings, a set of sensitivity analyses were conducted. First, a cost-utility analysis (CUA) (35) using complete cases was conducted to assess the impact of the missing data. Second, a CUA was performed from a NHS/PSS and education perspective to account for the economic impact from the education system. Third, a CUA was performed from a societal perspective to account for all the economic impact outside the NHS/PSS perspective, including parental productivity costs. Finally, a CUA that used the CHU-9D instead of the EQ-5D-Y to estimate QALYs based on the UK population tariff (19) was conducted to assess the impact of outcome measurement instrument.

All analyses were performed on an intention-to-treat basis using Stata version 16 ((StataCorp, College Station, Texas, USA).

2.7 Ethical approval and informed consent

This study was funded by the National Institute for Health Research (NIHR) Public Health Research (PHR) programme (PHR15/49/32), and the International Standard Randomised Controlled Trial Number is ISRCTN64852382 (2). Positive ethical opinion has been obtained via the University of York Research Ethics Committee (18/HRA/0101), and the written informed consent was obtained from parents on behalf of their child. Children assented to be part of the groups and did not proceed if they were not willing.

For peer review only - http://bmjopen.bmj.com/site/about/guidelines.xhtml

2.8 Patient and Public Involvement

No patient involved.

3. Results

3.1 Participants

A total of 284 CYP with ASD were recruited in the trial. After removing 34 ineligible CYP and 2 CYP who were not eligible for multiple imputation due to missing baseline utilities, 248 CYP with ASD were available for primary analysis (126 were allocated to LEGO® based therapy and 122 to usual support). This sample constitutes the base-case group. On the other hand, only 139 (56.0%) CYP had both EQ-5D and resource use (from the NHS and PSS perspective) data at all three data collection time points. This sample constitutes the complete-case group. Overall, 27.8% of cost or QALY measurements were missing and were imputed for primary analysis.

Descriptive statistics of CYP's baseline characteristics for both complete-case and base-case are presented in Table 1. As shown, more than third-quarters of the CYP in the LEGO® based therapy and the usual care arms were male, and more than 50% of the CYP in both arms were in primary school age (ranging from 7 to 11 years old). Differences in the SCQ scores at the baseline were marginal across arms and samples. Overall, the baseline characteristics are consistent across samples (base-case and complete-case).

Table 1: Baseline characteristics by trial arm

	Base case (n=248)		Complete case (n=139)	
**Baseline characteristics**	**LEGO® based therapy (N=126)**	**Usual support (N=122)**	**LEGO® based therapy (N=80)**	**Usual support (N=59)**
Gender, n (%)				
Male	101 (80.2%)	91 (74.6%)	68 (85.0%)	43 (72.9%)
Age (years), n (%)				
7-11	83 (65.9%)	79 (64.8%)	54 (67.5%)	43 (72.9%)
11-15	43 (34.1%)	43 (35.2%)	26 (32.5%)	16 (27.1%)
Mean (sd)	9.7 (2.3)	9.8 (2.2)	9.6 (2.2)	9.6 (2.2)
Year from diagnosis				
Mean (sd)	3.4 (2.7)	3.6 (2.8)	3.2 (2.4)	3.6 (3.0)
Social Communication Questionnaire (SCQ) scores				
Mean (sd)	25.1 (5.2)	24.2 (5.2)	24.9 (5.1)	24.1 (5.0)
EQ-5D				
Mean (sd)	0.79 (0.11)	0.76 (0.11)	0.79 (0.12)	0.77 (0.11)
Site, n (%)				
Leeds	37 (29.4%)	38 (31.2%)	31 (38.8%)	18 (30.5%)
Sheffield	70 (55.6%)	67 (54.9%)	34 (42.5%)	31 (52.5%)

For peer review only - http://bmjopen.bmj.com/site/about/guidelines.xhtml

York	19 (15.1%)	17 (13.9%)	15 (18.7%)	10 (17.0%)
Number of intervention sessions				
Mean (sd)	10.3 (2.3)	-	10.5 (2.2)	-

3.2 Costs

On average, the estimated intervention cost per session per CYP was £6.5 (£2.45 for training and £4.05 for intervention delivery). The main cost driver of training costs was trainer fees, while the main cost drivers of intervention delivery costs were the costs for preparation and delivery the intervention, and the costs for LEGO® materials (Appendix 2).

In terms of service costs, the average total service costs over 52 weeks to the NHS providers (after imputation) were £524 (95%CI: £428 to 808) for the LEGO® based therapy arm compared with £678 (95% CI: £427 to 928) for the usual care arm. The cost difference is larger when the societal perspective was considered, as CYP in the LEGO® based therapy arm incurred less costs across all the perspectives. The largest cost differences occurred in education related services followed by healthcare and social services. It is worth noting that some of cost differences were likely to have been driven by high-cost cases. For instance, in complete-case, higher average cost of Child and Adolescent Mental Health Services (CAMHS)-related services in the usual care arm was driven by two high-cost cases, and higher average cost of school-based health related services in the LEGO® based therapy arm was driven by one high-cost case. These high-cost cases were kept in the analysis, as they were plausible. However, due to the high-cost cases, the cost differences need to be interpreted with caution. A more detailed overview on the service costs over 52 weeks and the resource use are presented in Table 2 and Appendix 3, respectively.

For peer review only

For peer review only - http://bmjopen.bmj.com/site/about/guidelines.xhtml

Table 2: Average costs of service use in one year by trial arm

	Base case		Complete case	
	**LEGO® based therapy (n=126), £ (95% CI)**	**Usual support (n=122), £ (95% CI)**	**LEGO® based therapy (n=80), £ (95% CI)**	**Usual support (n=59), £ (95% cI)**
**NHS and PSS**	**524 (372, 675)**	**678 (427, 928)**	**618 (428, 808)**	**752 (420, 1,083)**
Community-based services				
CAMHS related	77 (40, 114)	233 (37, 428)	117 (50, 184)	267 (19, 516)
Non-CAMHS related	115 (79, 151)	99 (69, 130)	120 (78, 161)	107 (67, 148)
Hospital-based services				
Mental health related	20 (6, 33)	45 (11, 79)	19 (4, 33)	53 (-1, 107)
Non-mental health related	60 (29, 92)	86 (37, 136)	79 (30, 128)	89 (31, 147)
Medications				
Mental health related	195 (115, 275)	129 (74, 185)	211 (121, 301)	142 (75, 208)
Non-mental health related	57 (18, 97)	85 (25, 145)	73 (22, 124)	93 (19, 167)
**Education system related**	**1,204 (949, 1,458)**	**1,437 (1,082, 1,792)**	**1,388 (989, 1,787)**	**1,633 (1,041, 2,224)**
School-based health	164 (62, 267)	88 (20, 156)	182 (48, 316)	100 (13, 186)
Intervention support	712 (496, 927)	948 (645, 1,250)	793 (458, 1,128)	1,070 (615, 1,526)
General support	327 (242, 413)	401 (262, 541)	368 (245, 492)	473 (237, 709)
**Private expenses**	**211 (129, 293)**	**317 (189, 445)**	**192 (105, 280)**	**329 (171, 487)**
Childcare	211 (129, 293)	317 (189, 445)	192 (105, 280)	329 (171, 487)
**Productivity**	**95 (57, 132)**	**114 (64, 164)**	**104 (62, 146)**	**111 (53, 170)**
Parental productivity loss	95 (57, 132)	114 (64, 164)	104 (62, 146)	111 (53, 170)
**Total costs**	**2,033 (1,710, 2,357)**	**2,546 (2,087, 3,005)**	**2,278 (1,775, 2,781)**	**2,819 (2,123, 3,515)**

Community-based services: health services provided outside of a hospital setting, including services provided by GPs, by applied health professionals (community nurse, community paediatrician, occupational therapist, physiotherapist, and Speech and Language therapist for non-CAMHS related services, and child psychiatrist, child psychotherapist, child psychologist, clinical psychologist, mental health nurse, and Primary mental health worker for CAMHS related services) and by social services (social care worker, home care worker, family support worker, and Helpline (e.g. Samaritans))
CAMHS: Child and Adolescent Mental Health Services, including child psychiatrist, child psychotherapist, child psychologist, clinical psychologist, mental health nurse, and primary mental health worker (PMHW)
Hospital-based services: health services provided in a hospital setting, including inpatient stays, outpatient visits and emergency services.
Childcare included paid childcare, after school club, religious club, and special clubs for autism children

For peer review only - http://bmjopen.bmj.com/site/about/guidelines.xhtml

For peer review only

3.3 Outcomes

Table 3 shows the mean EQ-5D-Y (3L proxy) and CHU-9D utility scores between the two arms of the trial at each time point when scores were not imputed (complete case) and when scores were imputed (base case). As shown, in both arms, there was no significant change in EQ-5D-Y or CHU-9D utility scores from baseline to 52 weeks. The fluctuations between baseline and 20 weeks, and between 20 weeks and 52 weeks, were small in both the base and the complete cases. After calculation using the area under the curve approach, it is found that the LEGO® based therapy produced marginally higher mean QALYs (0.03 QALYs) compared to the usual support regardless of the instrument used. Further details for the responses of EQ-5D and CHU-9D in each domain can be found in Appendix 4 and Appendix 5, respectively.

Table 3: Average EQ-5D-Y and CHU-9D utility scores by trial arm

	Base case		Complete case	
**Time point**	**LEGO® based therapy (n=126), mean (95% CI)**	**Usual support (n=122) mean (95% CI)**	**LEGO® based therapy (n=80), mean (95% CI)**	**Usual support (n=59) mean (95% CI)**
EQ-5D-Y				
Baseline	0.79 (0.77, 0.81)	0.76 (0.74, 0.79)	0.79 (0.77, 0.81)	0.77 (0.74, 0.79)
20 weeks	0.78 (0.76, 0.81)	0.76 (0.74, 0.78)	0.79 (0.76, 0.81)	0.75 (0.74, 0.79)
52 weeks	0.79 (0.76, 0.81)	0.76 (0.74, 0.79)	0.80 (0.77, 0.82)	0.75 (0.73, 0.80)
Total QALYs	0.79 (0.77, 0.80)	0.76 (0.74, 0.78)	0.79 (0.77, 0.81)	0.76 (0.74, 0.79)
CHU-9D				
Baseline	0.83 (0.80, 0.85)	0.81 (0.79, 0.84)	0.84 (0.81, 0.87)	0.83 (0.80, 0.86)
20 weeks	0.84 (0.82, 0.86)	0.80 (0.78, 0.83)	0.83 (0.80, 0.86)	0.78 (0.74, 0.82)
52 weeks	0.83 (0.81, 0.85)	0.80 (0.77, 0.83)	0.81 (0.78, 0.84)	0.81 (0.77, 0.85)
Total QALYs	0.83 (0.82, 0.85)	0.80 (0.78, 0.82)	0.82 (0.80, 0.85)	0.80 (0.76, 0.83)

3.4 Cost-utility analysis (primary analysis)

After accounting for uncertainty and unbalanced baseline utility and characteristics, on average, CYP with ASD receiving LEGO® based therapy incurred £251 (95% CI - £268 to £752) less costs from the NHS/PSS perspective and gained 0.009 (95% CI -0.008 to 0.028) extra QALYs measured by EQ-5D-Y than those having usual support. The bootstrapped ICER results are presented in Figure 1A, and the probabilities of LEGO® based therapy being cost-effective over a range of WTP threshold are presented in Figure 1B. As shown, the simulated estimates were largely below the threshold line, and the probability of LEGO® based therapy being cost-effective is 94% at the WTP threshold of £20,000. The findings

For peer review only - http://bmjopen.bmj.com/site/about/guidelines.xhtml

suggest that the LEGO® based therapy was likely to be cost-effective, although the incremental costs and QALYs were marginal.

3.5 sensitivity analysis

Results of sensitivity analyses are detailed in Appendix 6. The mean incremental cost and QALY estimates from the complete-case were along the line of the based-case scenario, yielding a negative cost per QALY gained. Sensitivity analyses using a societal perspective to measure costs and the CHU-9D to measure QALYs were also conducted. In both sensitivity analyses, the ICER pairs lay below the recommended NICE threshold (£20,000-30,000/QALY gained), and the majority of the bootstrapped estimates sat in the bottom right quadrant (Figure 2), suggesting that LEGO® based therapy was likely to be cost-effective.

4. Discussion

4.1 Principal findings

To the best of our knowledge, this is the first study evaluating the cost-effectiveness of LEGO® based therapy for CYP with ASD. Compared to usual care, the LEGO® based therapy marginally decreased the service use costs and increased the QALYs from the NHS/PSS perspective. This is evident in both primary and sensitivity analyses, which considered costs derived from various perspectives and QALYs measured by different instruments.

4.2 Implications of study

The average QALYs measured by both the EQ-5D-Y (proxy version) and the CHU9D for those in the intervention arm were higher compared to the control arm. Although the differences are small, it is observed that the bootstrap estimates plotted on the CE planes for both primary and sensitivity analyses were mainly in the bottom right quadrant. This indicates that after taking uncertainty into consideration and adjusting for the imbalanced baseline utility, the differences in QALYs remain positive. It is also worth noting that such differences were unaffected regardless who filled the questionnaire. Both the parent-completed (EQ-5D) and CYP-completed (CHU9D) questionnaires showed the same positive differences.

The study also shows a reduction in average total NHS/PSS costs (albeit with wide confidence intervals), particularly through attendance at Child and Adolescent Mental Health Services (CAMHS). As mentioned in section 3.2, the lowered CAMHS costs found in the LEGO® based therapy arm was more related to a small number of CYP receiving high levels of high-tariff CAMHS support in the control arm (usual support) than the intervention arm rather than a general drop across the whole group. Such a finding indicates that CYP who had co-occurring emotional and behavioural problems seemed to be receiving

For peer review only - http://bmjopen.bmj.com/site/about/guidelines.xhtml

approximately similar support from CAMHS across both trial arms, and LEGO® based therapy did not overshadow the needs of CAMHS support. It was also found that, at the baseline, CYP in LEGO® based therapy arm had higher frequency of CAMHS support compared to control arm. Given the high threshold for receiving CAMHS support, it is likely that the CYP in LEGO® based therapy arm had more severe needs at baseline. However, after the intervention, CYP in LEGO® based therapy arm had similar support from CAMHS as those in control arm (see above) suggesting some amelioration effect or a reduced need for such high level support. Based on the literature of general research on CAMHS referrals, such reduction could be because the LEGO® based therapy improves co-morbid problems of CYP and consequently leads to a reduced likelihood of referral to CAMHS (37) or stops the CYP being seen by CAMHS (38). However, it is also possible that the school-based LEGO® based therapy improves a CYP's social skills and leads to less distress or conflict and may subsequently reduce the likelihood for referral to CAMHS (39) because of reduced levels of teacher and parent/guardian concern. Research to date, however, suggests higher parental anxiety tends to reduce referral rates not increase them in many circumstances (40).

The reduction in school intervention costs was also observed in this study. One possible explanation is that CYP in a LEGO® based therapy intervention might be less likely to be put forward for other interventions (e.g., the social use of language programme (SULP) (41)). Another possible reason might include a belief by a parent/guardian that an active intervention is happening, and so for this reason taking part in another intervention at the same time is not necessary. While both possible explanations might be valid, there was evidence that schools reported a wide range of other interventions being received by CYP with ASD, including Social Stories™, visual schedules, 1:1 mentoring, and others. Whether LEGO® based therapy reduced certain type of interventions but increases other type of interventions remains unclear. Further investigations would be needed to explore this.

Finally, the bootstrapping results on the cost-effectiveness plane (Figure 1A and 2) demonstrate the dispersion of iterations. It is observed that the confidence intervals for incremental costs and incremental QALYs were wide, both in primary and sensitivity analyses. This indicated high levels of uncertainty around the estimate of the incremental costs and QALYs and, consequently, wide confidence intervals of the estimated ICERs. The phenomenon may be due to the small average cost reduction and small mean QALY gained, but large variation among the CYP. This could be also because the EQ-5D-Y instrument can be less responsive or sensitive to small changes in mental health (42). Although the confidence intervals for the ICERs were wide, the LEGO® based therapy remains highly likely to be cost-effective, as the majority of cost-QALY pairs were below the £20,000 threshold (Figure 1 and 2).

For peer review only - http://bmjopen.bmj.com/site/about/guidelines.xhtml

4.3 Strengths and limitations

This is the first study evaluating the cost-effectiveness of LEGO® based therapy and one of only a few economic evaluation studies for CYP with ASD (8,9). Such a study is important because there is a growing popularity of LEGO® based therapy in the UK. Furthermore, since detailed resource use in school was able to be collected via teacher-completed questionnaire, our study managed to capture the cost difference in school in a more granular manner and reflect the reality in school better. Additionally, our study accounts for the costs measured from a range of perspectives and the QALYs measured by different instruments. The approach not only ensures the robustness of our findings but also can help policy makers from different sectors to make informed decision. This is particularly true in the UK setting, as organisations such as the Department for Education (DfE), the Department of Health and Social Care (DH), and the local authorities are working together to ensure CYP with special educational needs and disabilities (SEND) properly supported based on the SEND Code of Practice 2014 (43) and the Children and Families Act 2014 (44). Some other considerations beyond cost effectiveness, such as acceptability and equality of access may be also taken into account by decision makers. Findings of strong acceptability to schools, children, CYP and their families were reported elsewhere (45), whereas equality of access would need further exploration in the future, as at present we do not have sufficient data to undertake any form of statistical analysis. Finally, this study has a large sample size compared to other similar trials in ASD (8,46) and is one of the few ASD intervention studies to date follow up to one year (8,47). This would make our results more generalizable and robust compared to the similar study with small sample size or shorter term follow-up.

Despite the strengths mentioned above, our study was subject to a few limitations. Firstly, funding sources for a few types of staff was not always clear. For example, speech and language therapists can be funded by NHS, schools or local authorities. Such diversity causes difficulties when it comes to costing and reporting the results, as detailed information about funders for each member of staff involved was unavailable. Several assumptions have been made based on service locations and published guidelines (i.e. the unit costs of Health and Social Care from PSSRU 2018) for costing. Hence, the summarised cost results for different perspectives need to be treated with caution. However, both arms were treated the same way and the overall costs (from the societal perspective) should remain robust. Secondly, a small number of high but plausible values were observed in cost estimations. Although the values did not affect the cost-effectiveness conclusions (data not shown), such values can potentially bias the cost reduction results of LEGO® based therapy. Hence, care should be taken when interpreting the cost estimates. It is none-the-less important to include this real world data and be aware of this for future studies. Thirdly, the calculated intervention costs

For peer review only - http://bmjopen.bmj.com/site/about/guidelines.xhtml

For peer review only

might have been underestimated. This is because several items associated with training and intervention sessions were not costed, due to data constraints. These included opportunity costs of trainee time, opportunity costs of school venue for delivering interventions, recruitment cost if intervention rolled out, and supervision costs. However, this is unlikely to have affected the results of the dominance of the intervention over usual support, as these costs are considered to be small. This is especially the case after the allocating to every CYP for every session. Further research on the exploration and measurement of the costs with considerations is desirable. Finally, our study used SURE to model the uncertainty around the incremental costs and QALYs and account for their correlation. Alternatively, costs and QALYs can be modelled separately using generalised linear models without considering the correlation. It is beyond the scope of our study to compare the two methods. However, further research on the method comparison and their impacts on the results are desirable in order to draw robust conclusions.

4.4 Future work

Our study measured the short-term cost-effectiveness of LEGO® based therapy on CYP with ASD over one year follow-up. For the long-term cost-effectiveness of LEGO® based therapy, a modelled-based economic evaluation study would be desirable to allow life-time cost-effectiveness and children's lost productivity during adulthood to be measured. Further research is also needed on exploring potential impacts on other outcomes such as academic achievement or quality of life of other family members. In future research, it would be also helpful to explore whether a longer duration of intervention (e.g. a full school year) or more frequent sessions (e.g. twice a week) would further improve outcomes while remaining cost-effective.

5. Conclusion

This study demonstrates the potential cost-effectiveness of delivering LEGO® based therapy to CYP with ASD in mainstream school settings. The findings will be of interest to NHS health and social care providers, local authorities, families and community professionals including school staff members.

For peer review only - http://bmjopen.bmj.com/site/about/guidelines.xhtml

Figures

Figure 1: Cost-effectiveness plane and CEAC of primary analysis

Figure 2: Cost-effectiveness planes of sensitivity analyses

For peer review only

For peer review only - http://bmjopen.bmj.com/site/about/guidelines.xhtml

Authors' contributions

HW and SP were the trial health economists and analysed the data with HW taking the lead in writing the manuscript. BW, CC, ALC, DT, KB, GGDLC, DM, DV and SG conceived the study idea and designed the project. MB and DT were the study statisticians and involved in the analysis and interpretation of the data. EK and KM managed the trial, and TC, AB, KS, AP and RN were involved in the acquisition and management of the data. All authors contributed to data interpretation and have read and approved the final manuscript.

Competing interests

The research team was aware that the LEGO® name is a registered trademark and followed the fair use policy in regard to the LEGO® brand throughout the duration of the trial. **Gina Gomez de la Cuesta** co-authored the LEGO® based therapy manual which formed the basis of the intervention delivered in the trial. The co-authors of the manual have given us full permission to use the manual without license and to develop an abridged version. They have also stated their support for us in writing our own version and will become co-authors on any future publications. **Gina Gomez de la Cuesta** has also agreed for the team to adapt the fidelity checklist used in her previous study. **Gina Gomez de la Cuesta** is a Director of Play Included a community interest company that offers training and resources for interventions involving play bricks for children. We have provisional agreement with Jessica Kingsley Publishers who have expressed interest in publishing the abridged manual. However, we are not tied to them as a publisher. There are no other financial and/or competing interests to declare.

Funding statement

This work was supported by the NIHR under its Programme Grants for Public Health Research (PHR) (Grant Reference Number PHR15/49/32)

Data sharing statement

No additional data available. However, Stata code that used for this study is available from the corresponding author, Han-I Wang, upon reasonable request.

Acknowledgments

We gratefully thank all families participating in the study. We acknowledge invaluable support and guidance from our trial steering committee (Professor David Cottrell (University of Leeds), Karen Watson (PPI), Dr Sue Fletcher-Watson (University of Edinburgh), Dr Fiona Warren (University of Exeter), Dr Michael Morton (University of Glasgow), Alison Thompson (Leeds and York Partnership NHS Foundation Trust)), and our data monitoring and ethics committee (Dr Zoe Hoare (Bangor University), Dr David Simms (Bradford District Care NHS

For peer review only - http://bmjopen.bmj.com/site/about/guidelines.xhtml

Foundation Trust), John O'Dwyer (University of Leeds)). We also gratefully acknowledge the hard work, support and advice from the following:

PPI representatives

The Young Dynamos research advisory group, the National Autistic Society, Karen Watson (PPI TSC member), the York Youth Council and several individuals including Tina Hardman and Ann LcLaren.

Participant screening and data collection

Sarah Jacob-Eshtan, Lisa Hackney, Rebecca Hargate, Sam Bennett, Emma Sellers, Holly Taylor, Alix Smith, Katie Sutherland, Richard Campbell, Rachel Hodkinson, Megan Garside, Jane Blackwell, Pavithra Kumar, Jennifer Lomas

Administrative and clerical support

Sharon Bird, Rita Lynch, Daniel Gottschalk, Katy Harmston, Thasamia Akhtar, Louise Turner, Heather Dakin

Sponsor Study monitoring

Tahir Idrees

LEGO® based therapy expertise

Elinor Brett, Abigail Dodson

Support on data management concerns

Emily Turton

Support on ethical and governance issues.

Sinead Audsley, Stephen Holland

The authors would also like to thank Daniel LeGoff for information about LEGO® based therapy in the USA and both the Leeds and York Partnership NHS Foundation Trust and the University of York for supporting the Child Oriented Mental Health Intervention Centre (COMIC) and the University of Sheffield CTRU in taking this research from an idea to a full trial.

The research team are aware that the LEGO® name is a registered trademark and will follow the fair use policy in regard to the LEGO® brand throughout the duration of the trial. The team have been in discussion with LEGO, and they have agreed for the use of this term for the project and its outputs, but not over the longer term. After PPI work, we have paired the term LEGO® based therapy with the new term Play Brick Therapy, which we suggest is used henceforth.

Disclaimer

The views and opinions expressed by authors in this publication are those of the authors and do not necessarily reflect those of the NHS, the NIHR, NETSCC, the PHR programme or

For peer review only

the Department of Health and Social Care.

Patient consent form

Not required.

Ethical approval

Ethics approval has been obtained via the University of York Research Ethics Committee (18/HRA/0101)

For peer review only

For peer review only - http://bmjopen.bmj.com/site/about/guidelines.xhtml

Reference

1. American Psychiatric Association. Diagnostic and Statistical Manual of Mental Disorders (DSM–5). [Internet]. 5th edition. Arlington: American Psychiatric Association; 2013. Available from: https://www.psychiatry.org/psychiatrists/practice/dsm

2. Baron-Cohen S, Scott FJ, Allison C, Williams J, Bolton P, Matthews FE, et al. Prevalence of autism-spectrum conditions: UK school-based population study. Br J Psychiatry J Ment Sci. 2009 Jun;194(6):500–9.

3. Baird G, Simonoff E, Pickles A, Chandler S, Loucas T, Meldrum D, et al. Prevalence of disorders of the autism spectrum in a population cohort of children in South Thames: the Special Needs and Autism Project (SNAP). Lancet Lond Engl. 2006 Jul 15;368(9531):210–5.

4. Office for National Statistics. 2011 Census Aggregate Data. UK Data Service; 2017.

5. Buescher AVS, Cidav Z, Knapp M, Mandell DS. Costs of autism spectrum disorders in the United Kingdom and the United States. JAMA Pediatr. 2014 Aug;168(8):721–8.

6. Holmes EA, Ghaderi A, Harmer CJ, Ramchandani PG, Cuijpers P, Morrison AP, et al. The Lancet Psychiatry Commission on psychological treatments research in tomorrow's science. Lancet Psychiatry. 2018 Mar 1;5(3):237–86.

7. Sampaio F, Feldman I, Lavelle TA, Skokauskas N. The cost-effectiveness of treatments for attention deficit-hyperactivity disorder and autism spectrum disorder in children and adolescents: a systematic review. Eur Child Adolesc Psychiatry [Internet]. 2021 Mar 9 [cited 2021 Mar 26]; Available from: https://doi.org/10.1007/s00787-021-01748-z

8. Byford S, Cary M, Barrett B, Aldred CR, Charman T, Howlin P, et al. Cost-effectiveness analysis of a communication-focused therapy for pre-school children with autism: results from a randomised controlled trial. BMC Psychiatry. 2015 Dec 21;15:316.

9. Penner M, Rayar M, Bashir N, Roberts SW, Hancock-Howard RL, Coyte PC. Cost-Effectiveness Analysis Comparing Pre-diagnosis Autism Spectrum Disorder (ASD)-Targeted Intervention with Ontario's Autism Intervention Program. J Autism Dev Disord. 2015 Sep;45(9):2833–47.

10. Baron-Cohen S, Cuesta GGDL, LeGoff DB, Krauss GW. LEGO®-Based Therapy: How to build social competence through LEGO®-based Clubs for children with autism and related conditions. Jessica Kingsley Publishers; 2014. 146 p.

For peer review only

For peer review only - http://bmjopen.bmj.com/site/about/guidelines.xhtml

11. North Yorkshire County Council. North Yorkshire County Council Intervention Guidance 2019. [Internet]. 2019 [cited 2021 Apr 8]. Available from: https://cyps.northyorks.gov.uk/sites/default/files/SEND/Social,%20Emotional%20and%20Mental%20Health/76099_Intervention%20guidanc_proof%2001.pdf

12. Varley D, Wright B, Cooper C, Marshall D, Biggs K, Ali S, et al. Investigating SOcial Competence and Isolation in children with Autism taking part in LEGO-based therapy clubs In School Environments (I-SOCIALISE): study protocol. BMJ Open. 2019 May 1;9(5):e030471.

13. Wright B, Marshall D, Adamson J, Ainsworth H, Ali S, Allgar V, et al. Social StoriesTM to alleviate challenging behaviour and social difficulties exhibited by children with autism spectrum disorder in mainstream schools: design of a manualised training toolkit and feasibility study for a cluster randomised controlled trial with nested qualitative and cost-effectiveness components. Health Technol Assess (Rockv). 2016;20(6):1–258.

14. LeGoff DB. Use of LEGO as a therapeutic medium for improving social competence. J Autism Dev Disord. 2004 Oct;34(5):557–71.

15. Owens G, Granader Y, Humphrey A, Baron-Cohen S. LEGO therapy and the social use of language programme: an evaluation of two social skills interventions for children with high functioning autism and Asperger Syndrome. J Autism Dev Disord. 2008 Nov;38(10):1944–57.

16. Ravens-Sieberer U, Wille N, Badia X, Bonsel G, Burström K, Cavrini G, et al. Feasibility, reliability, and validity of the EQ-5D-Y: results from a multinational study. Qual Life Res Int J Qual Life Asp Treat Care Rehabil. 2010 Aug;19(6):887–97.

17. Stevens K. Developing a descriptive system for a new preference-based measure of health-related quality of life for children. Qual Life Res Int J Qual Life Asp Treat Care Rehabil. 2009 Oct;18(8):1105–13.

18. Dolan P. Modeling valuations for EuroQol health states. Med Care. 1997 Nov;35(11):1095–108.

19. Stevens K. Assessing the performance of a new generic measure of health-related quality of life for children and refining it for use in health state valuation. Appl Health Econ Health Policy. 2011 May 1;9(3):157–69.

20. Glick H, Doshi J, Polsky D, Sonnad S. Economic evaluation in clinical trials. second. Oxford: Oxford University Press; 2014.

For peer review only - http://bmjopen.bmj.com/site/about/guidelines.xhtml

21. Barrett B, Byford S, Chitsabesan P, Kenning C. Mental health provision for young offenders: service use and cost. Br J Psychiatry J Ment Sci. 2006 Jun;188:541–6.

22. Wright B, Marshall D, Moore DC, Ainsworth H, Hackney L, Adamson J, et al. Autism Spectrum Social Stories In Schools Trial (ASSSIST): study protocol for a feasibility randomised controlled trial analysing clinical and cost-effectiveness of Social Stories in mainstream schools. BMJ Open. 2014 Jul 1;4(7):e005952.

23. Marshall D, Wright B, Allgar V, Adamson J, Williams C, Ainsworth H, et al. Social Stories in mainstream schools for children with autism spectrum disorder: a feasibility randomised controlled trial. BMJ Open. 2016 Aug 11;6(8):e011748.

24. NHS Improvement. NHS reference costs 2018/19 [Internet]. [cited 2021 Mar 28]. Available from: https://www.england.nhs.uk/national-cost-collection/

25. Curtis L, Burns A. Unit Costs of Health and Social Care 2018 [Internet]. Canterbury: Univerity of Kent; 2018 [cited 2021 Mar 28]. Available from: https://www.pssru.ac.uk/project-pages/unit-costs/unit-costs-2018/

26. NHS Digital. Prescription Cost Analysis - England 2018 [Internet]. Health and Social Care Information Centre; 2019 [cited 2021 Mar 28]. Available from: https://digital.nhs.uk/data-and-information/publications/statistical/prescription-cost-analysis/2018

27. Family and Childcare Trust. Childcare Survey 2018 [Internet]. Family and Childcare; [cited 2021 Mar 28]. Available from: https://www.familyandchildcaretrust.org/childcare-survey-2018

28. Department for Education. DfE consolidated annual report and accounts 2017 to 2018 [Internet]. London; 2018 [cited 2021 Mar 28]. Available from: https://www.gov.uk/government/publications/dfe-consolidated-annual-report-and-accounts-2017-to-2018

29. National Agreement on Pay and Conditions for Service (Green Book). National Joint council; 2018 p. 225.

30. Employee earnings in the UK (2018) [Internet]. Office for National Statistics; 2019 [cited 2021 Mar 28]. Available from: https://www.ons.gov.uk/employmentandlabourmarket/peopleinwork/earningsandworkinghours/bulletins/annualsurveyofhoursandearnings/2018

31. Rubin DB. Statistical matching using file concatenation with adjusted weights and

For peer review only - http://bmjopen.bmj.com/site/about/guidelines.xhtml

multiple imputations. J Bus Econ Stat. 1986 Jan 1;4(1):87–94.

32. Manca A, Hawkins N, Sculpher MJ. Estimating mean QALYs in trial-based cost-effectiveness analysis: the importance of controlling for baseline utility. Health Econ. 2005 May;14(5):487–96.

33. Briggs AH, Wonderling DE, Mooney CZ. Pulling cost-effectiveness analysis up by its bootstraps: A non-parametric approach to confidence interval estimation. Health Econ. 1997;6(4):327–40.

34. Hoch JS, Briggs AH, Willan AR. Something old, something new, something borrowed, something blue: a framework for the marriage of health econometrics and cost-effectiveness analysis. Health Econ. 2002 Jul;11(5):415–30.

35. Fenwick E, O'Brien BJ, Briggs A. Cost-effectiveness acceptability curves--facts, fallacies and frequently asked questions. Health Econ. 2004 May;13(5):405–15.

36. National Institute of Health and Care Excellence (NICE). Guide to the methods of technology appraisal [Internet]. NICE; 2013 [cited 2021 Mar 28]. Available from: https://www.nice.org.uk/process/pmg9/chapter/foreword

37. Costello EJ, Janiszewski S. Who gets treated? Factors associated with referral in children with psychiatric disorders. Acta Psychiatr Scand. 1990 Jun;81(6):523–9.

38. Miller LM, Southam-Gerow MA, Allin RB. Who Stays in Treatment? Child and Family Predictors of Youth Client Retention in a Public Mental Health Agency. Child Youth Care Forum. 2008 Aug 1;37(4):153–70.

39. Sayal K. Annotation: Pathways to care for children with mental health problems. J Child Psychol Psychiatry. 2006 Jul;47(7):649–59.

40. Jongerden L, Simon E, Bodden DHM, Dirksen CD, Bögels SM. Factors associated with the referral of anxious children to mental health care: the influence of family functioning, parenting, parental anxiety and child impairment. Int J Methods Psychiatr Res. 2015 Mar;24(1):46–57.

41. Rinaldi W. Social Use of Language Programme. Infant and primary school teaching pack. Cranleigh: Wendy Rinaldi.; 2004.

42. Brazier, J. (2010). Is the EQ–5D fit for purpose in mental health? British Journal of Psychiatry, 197(5), 348-349.

For peer review only - http://bmjopen.bmj.com/site/about/guidelines.xhtml

43. Special educational needs and disability code of practice: 0 to 25 years. Deaprtment for Education and Department of Health; 2014 [cited 2021 October 28]. Available from: https://assets.publishing.service.gov.uk/government/uploads/system/uploads/attachment_data/file/398815/SEND_Code_of_Practice_January_2015.pdf

44. Children and Families Act 2014. [cited 2021 October 28]. Availale from: https://www.legislation.gov.uk/ukpga/2014/6/contents/enacted

45. Barr A, Coates E, Kingsley E, Biggs K, Le Couteur A, Wright B (2021) A mixed methods evaluation of the acceptability of therapy using LEGO® bricks (LEGO® based therapy) in mainstream primary and secondary education (submitted to Autism Research)

46. Strydom A, Bosco A, Vickerstaff V, Hunter R, Poppe M, Ratti V, et al. Clinical and cost effectiveness of staff training in the delivery of Positive Behaviour Support (PBS) for adults with intellectual disabilities, autism spectrum disorder and challenging behaviour - randomised trial. BMC Psychiatry. 2020 Apr 15;20(1):161.

47. LeGoff DB, Sherman M. Long-term outcome of social skills intervention based on interactive LEGO© play. Autism. 2006 Jul 1;10(4):317–29.

For peer review only

For peer review only - http://bmjopen.bmj.com/site/about/guidelines.xhtml

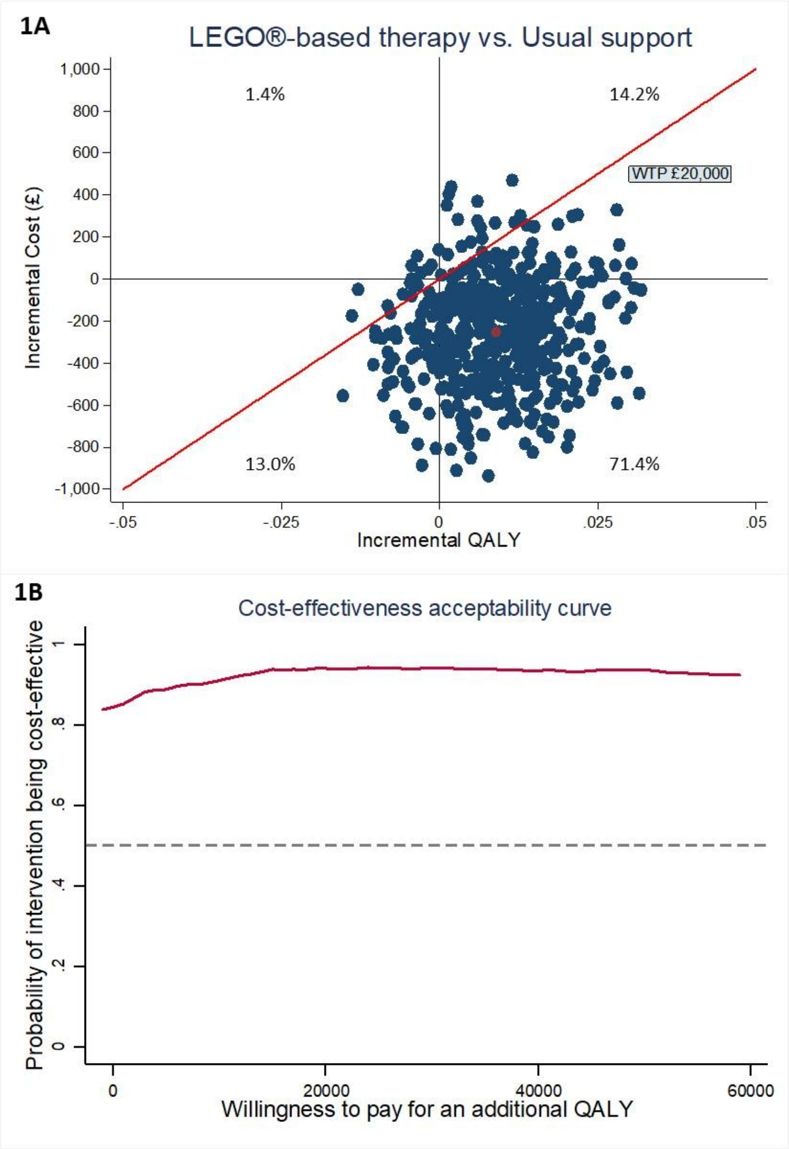

Figure 1: Cost-effectiveness plane and CEAC of primary analysis

Cost-effectiveness plane and CEAC of primary analysis

142x212mm (150 x 150 DPI)

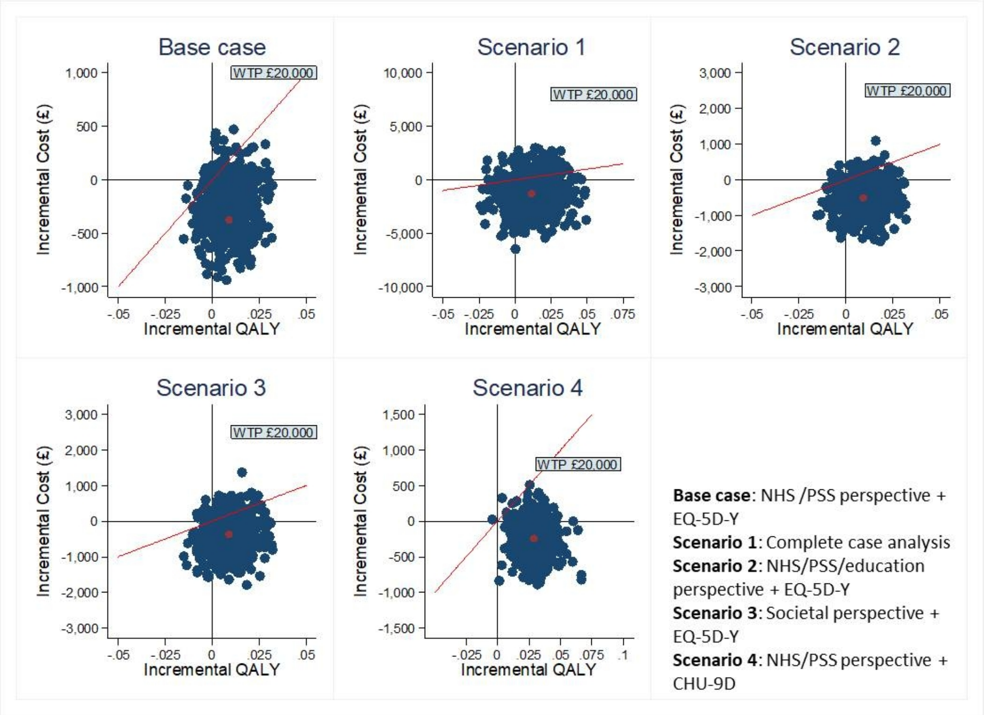

Figure 2: Cost-effectiveness planes of sensitivity analyses

Cost-effectiveness planes of sensitivity analyses

90x70mm (300 x 300 DPI)

Appendix 1: Inclusion and exclusion criteria

Inclusion criteria:

A participant was included if the CYP:

- Was aged between 7 and 15 years at the time of randomisation of the school.
- Attended a mainstream school in years two to 10.
- The CYP and parent/guardian had a sufficient understanding of English to be able to provide informed assent/informed consent (as appropriate) and read the LEGO®-based therapy instructions.
- Had an ASD clinical diagnosis from a qualified assessing clinician or team [based on best-practice guidance leading to an ICD-10 or DSM-5 diagnosis as reported by the CYP's parent/guardian and in the CYP's school records (this may have included the school's special educational needs (SEN) register, an individual education plan (IEP), individual health care plan, my support plan (MSPs), education health care plans (EHCPs), individual learning plans (ILP's) or equivalent).
- Had the ability to follow and understand simple instructions (as determined by the associated teacher/TA or parent/guardian).
- Scored 15 or higher on the Social Communication Questionnaire.

A school was included if:

- It was a mainstream school located in Leeds, York, Sheffield or surrounding areas in the North of England. This excludes specialist and independent schools.
- It had not used LEGO®-based therapy with the CYP in the current or preceding school term. For research purposes, LEGO®-based therapy was defined as meeting all of the main fidelity checklist criteria.
- They had at least one CYP diagnosed with ASD (in line with CYP inclusion criteria above)

Exclusion criteria:

A participant (CYP) was not eligible to take part in the study if:

- They had physical impairments which would prevent them participating in the activities (as assessed by the associated teacher/TA).

For peer review only - http://bmjopen.bmj.com/site/about/guidelines.xhtml

Appendix 2: Flow diagram

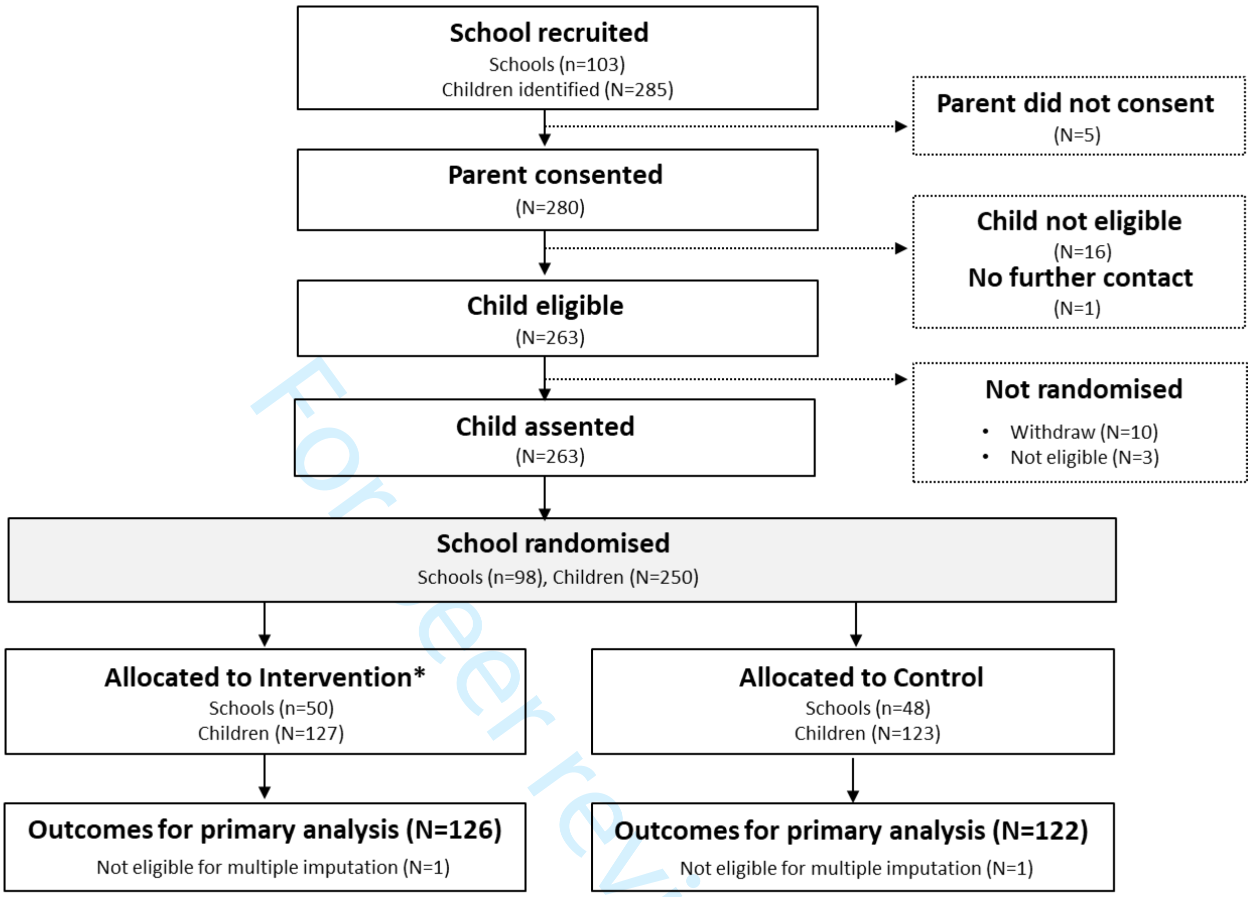

*following ITT principles, school (n=1) children (N-3) allocated to control, but received intervention are included in the control arm

For peer review only - http://bmjopen.bmj.com/site/about/guidelines.xhtml

Appendix 3: Intervention costs by trial arm

	Total cost (£)	Cost per session per child (£)
Training costs		
Trainer fee	£4,262	£2.01
Refreshment costs	£10	£0.00
Consumable costs	£178	£0.08
Trainer's travel costs	£740	£0.35
Total	£5,685	£2.45
Intervention costs		
LEGO	£3,903	£1.84
Intervention	£4,027	£1.90
Additional help	£77.3	£0.04
Consumables	£580.5	£0.27
Total	£8,587.8	£4.05

For peer review only

For peer review only - http://bmjopen.bmj.com/site/about/guidelines.xhtml

Appendix 4: Average service use by trial arm (complete case, n=139)

		Baseline		0-20 weeks		20-52 weeks	
	Unit	LEGO®-based therapy, N=80 Mean (sd)	Usual care, N=59 Mean (sd)	LEGO®-based therapy. N=80 Mean (sd)	Usual support N=59 Mean (sd)	LEGO®-based therapy, N=80 Mean (sd)	Usual support N=59 Mean (sd)
**NHS and PSS**							
Community-based services							
CAMHS related	Session	0.70 (2.76)	0.31 (0.88)	0.23 (0.88)	0.36 (1.06)	0.63 (2.81)	0.54 (2.46)
Non-CAMHS related							
GP	Appointment	0.45 (0.97)	0.24 (0.50)	0.39 (1.02)	0.22 (0.49)	0.71 (1.41)	0.66 (0.96)
Allied health professionals	Appointment	0.44 (0.93)	0.68 (2.20)	0.43 (1.00)	0.34 (0.71)	0.78 (1.96)	0.69 (1.56)
Social care services	Appointment	0.49 (1.65)	0.47 (1.34)	0.26 (1.00)	0.39 (2.11)	0.99 (2.62)	0.80 (2.06)
Hospital-based services / acute services							
Emergency services	Visit	0.19 (0.80)	0.12 (0.46)	0.13 (0.43)	0.03 (0.26)	0.20 (0.60)	0.17 (0.62)
Inpatient stay							
Mental health related	Night	-	-	-	-	-	-
Non-mental health related	Night	0.01 (0.11)	-	0.01 (0.11)	-	-	-
Outpatient visit / day case							
Mental health related	Visit	0.06 (0.37)	0.10 (0.44)	0.08 (0.38)	0.07 (0.31)	0.05 (0.22)	0.29 (1.37)
Non-mental health related	Visit	0.31 (1.71)	0.17 (0.59)	0.19 (0.75)	0.24 (0.63)	0.09 (0.40)	0.24 (0.95)
Medication							
Mental health related	Type	0.36 (0.80)	0.20 (0.48)	0.43 (0.81)	0.27 (0.55)	0.48 (0.87)	0.32 (0.51)
Non-mental health related	Type	0.33 (0.62)	0.41 (0.93)	0.41 (0.90)	0.53 (1.33)	0.38 (0.79)	0.59 (1.23)
**Education system related**							

For peer review only - http://bmjopen.bmj.com/site/about/guidelines.xhtml

School-based health	Hour	2.60 (10.00)	2.17 (6.94)	1.40 (5.80)	3.12 (10.51)	8.80 (32.99)	2.17 (10.06)
General support*	Hour	8.54 (7.26)	7.93 (12.68)	7.48 (9.25)	9.37 (17.34)	18.94 (30.32)	27.32 (45.13)
Intervention support*	Hour	5.50 (10.50)	4.66 (6.74)	6.78 (8.28)	7.15 (11.03)	8.37 (7.97)	7.72 (10.56)
**Private expanses – out of pocket**							
Childcare	Session	3.05 (7.35)	4.10 (11.05)	4.14 (9.30)	5.98 (12.37)	5.86 (13.58)	12.29 (25.20)
**Productivity**							
Parental productivity	Day	0.45 (1.18)	0.31 (0.93)	0.45 (1.04)	0.52 (1.51)	0.58 (1.13)	0.59 (1.40)

CAMHS: Child and Adolescent Mental Health Services, including child psychiatrist, child psychotherapist, child psychologist, clinical psychologist, mental health nurse, family therapist, and Primary mental health worker (PMHW)

Allied health professionals included community nurse, community paediatrician, occupational therapist, physiotherapist, and Speech and Language therapist

Social care services included social care worker, home care worker, family support worker, drug and alcohol support worker, and Helpline (e.g. Samaritans)

Childcare included paid childcare, after school club, religious club, and special clubs for autism children

*based on 117 teacher-reported questionnaires (68 from I-socialise arm and 48 from usual care arm)

For peer review only - http://bmjopen.bmj.com/site/about/guidelines.xhtml

Appendix 5: EQ-5D-Y responses by trial arms by data collection time points

Usual support (n=59)	Baseline			20 Weeks			52 Weeks		
	Level 1<br>n (%)	Level 2<br>n (%)	Level 3<br>n (%)	Level 1<br>n (%)	Level 2<br>n (%)	Level 3<br>n (%)	Level 1<br>n (%)	Level 2<br>n (%)	Level 3<br>n (%)
Mobility	52 (88.1)	7 (11.9)	-	46 (78.0)	13 (22.0)	-	46 (78.0)	13 (22.0)	-
Self-care	17 (28.8)	32 (54.2)	10 (17.0)	16 (27.1)	37 (62.7)	6 (10.2)	14 (23.7)	35 (59.3)	10 (17.0)
Usual activity	25 (42.4)	29 (49.1)	5 (8.5)	25 (42.4)	27 (45.8)	7 (11.8)	22 (37.3)	30 (50.8)	7 (11.9)
Pain/discomfort	45 (76.3)	13 (22.0)	1 (1.7)	37 (62.7)	22 (37.3)	-	38 (64.4)	21 (35.6)	-
Anxiety/depression	15 (25.4)	35 (59.3)	9 (15.3)	16 (27.1)	34 (57.6)	9 (15.3)	18 (30.5)	26 (44.1)	15 (25.4)
**Lego-based therapy (n=80)**	**Baseline**			**20 Weeks**			**52 Weeks**		
	Level 1<br>n (%)	Level 2<br>n (%)	Level 3<br>n (%)	Level 1<br>n (%)	Level 2<br>n (%)	Level 3<br>n (%)	Level 1<br>n (%)	Level 2<br>n (%)	Level 3<br>n (%)
Mobility	66 (82.5)	11 (13.7)	3 (3.8)	61 (76.2)	16 (20.0)	3 (3.8)	64 (80.0)	13 (16.2)	3 (3.8)
Self-care	25 (31.3)	36 (45.0)	19 (23.7)	26 (32.5)	42 (52.5)	12 (15.0)	29 (36.3)	36 (45.0)	15 (18.7)
Usual activity	33 (41.2)	41 (51.2)	6 (7.5)	35 (43.7)	36 (45.0)	9 (11.3)	43 (53.7)	33 (41.3)	4 (5.0)
Pain/discomfort	64 (80.0)	14 (17.5)	2 (2.5)	63 (78.7)	13 (16.3)	4 (5.0)	59 (73.8)	19 (23.7)	2 (2.5)
Anxiety/depression	40 (50.0)	33 (41.3)	7 (8.7)	37 (46.2)	34 (42.5)	9 (11.3)	39 (48.7)	29 (36.3)	12 (15.0)

*Level 1: none, Level 2: some, Level 3: extreme

For peer review only - http://bmjopen.bmj.com/site/about/guidelines.xhtml

Appendix 6: CHU-9D responses by trial arms by data collection time points

**Usual support (n=45)**	**Baseline**					**20 weeks**					**52 weeks**				
	**Level 1**	**Level 2**	**Level 3**	**Level 4**	**Level 5**	**Level 1**	**Level 2**	**Level 3**	**Level 4**	**Level 5**	**Level 1**	**Level 2**	**Level 3**	**Level 4**	**Level 5**
Worried	20 (44.4)	16 (35.6)	7 (15.6)	-	2 (4.4)	22 (48.9)	12 (26.7)	5 (11.1)	3 (6.7)	3 (6.7)	26 (57.8)	13 (28.9)	1 (2.2)	3 (6.7)	2 (4.4)
Sad	32 (71.1)	8 (17.8)	2 (4.4)	2 (4.4)	1 (2.2)	29 (64.4)	6 (13.3)	5 (11.1)	4 (8.9)	1 (2.2)	34 (75.6)	4 (8.9)	2 (4.4)	1 (2.2)	4 (8.9)
Annoyed	27 (60.0)	13 (28.9)	4 (8.9)	-	1 (2.2)	27 (60.0)	10 (22.2)	5 (11.1)	-	3 (6.7)	27 (60.0)	10 (22.2)	5 (11.1)	2 (4.4)	1 (2.2)
Tired	9 (20.0)	15 (33.3)	8 (17.8)	5 (11.1)	8 (17.8)	12 (26.7)	13 (28.9)	9 (20.0)	5 (11.1)	6 (13.3)	10 (22.2)	16 (35.6)	6 (13.3)	6 (13.3)	7 (15.6)
Pain	28 (62.2)	6 (13.3)	6 (13.3)	3 (6.7)	2 (4.4)	21 (46.7)	11 (24.4)	4 (8.9)	2 (4.4)	7 (15.6)	24 (53.3)	12 (26.7)	7 (15.6)	-	2 (4.4)
Sleep	21 (46.7)	12 (26.7)	5 (11.1)	3 (6.7)	4 (8.9)	20 (44.4)	3 (6.7)	7 (15.6)	9 (20.0)	6 (13.3)	17 (37.8)	16 (35.6)	4 (8.9)	4 (8.9)	4 (8.9)
Daily routine	23 (51.1)	12 (26.7)	4 (8.9)	3 (6.7)	3 (6.7)	19 (42.2)	11 (24.4)	7 (15.6)	4 (8.9)	4 (8.9)	24 (53.3)	10 (22.2)	5 (11.1)	3 (6.7)	3 (6.7)
Work	27 (60.0)	15 (33.3)	2 (4.4)	1 (2.2)	-	23 (51.1)	11 (24.4)	7 (15.6)	1 (2.2)	3 (6.7)	28 (62.2)	7 (15.6)	7 (15.6)	1 (2.2)	2 (4.4)
Able to join activities	23 (51.1)	6 (13.3)	10 (22.2)	3 (6.7)	3 (6.7)	10 (22.2)	14 (31.1)	10 (22.2)	9 (20.0)	2 (4.4)	20 (44.4)	10 (22.2)	10 (22.2)	1 (2.2)	4 (8.9)
**Lego-based therapy (n=51)**	**Baseline**					**20 weeks**					**52 weeks**				
	**Level 1**	**Level 2**	**Level 3**	**Level 4**	**Level 5**	**Level 1**	**Level 2**	**Level 3**	**Level 4**	**Level 5**	**Level 1**	**Level 2**	**Level 3**	**Level 4**	**Level 5**
Worried	34 (66.7)	8 (15.7)	3 (5.9)	2 (3.9)	4 (7.8)	33 (64.7)	6 (11.8)	7 (13.7)	1 (2.0)	4 (7.8)	34 (66.7)	7 (13.7)	6 (11.8)	3 (5.9)	1 (2.0)
Sad	40 (78.4)	5 (9.8)	2 (3.9)	1 (2.0)	3 (5.9)	37 (72.5)	5 (9.8)	5 (9.8)	1 (2.0)	3 (5.9)	36 (70.6)	6 (11.8)	6 (11.8)	2 (3.9)	1 (2.0)
Annoyed	37 (72.5)	7 (13.7)	4 (7.8)	2 (3.9)	1 (2.0)	34 (66.7)	10 (19.6)	5 (9.8)	-	2 (3.9)	37 (72.5)	8 (15.7)	3 (5.9)	1 (2.0)	2 (3.9)
Tired	14 (27.5)	19 (37.3)	5 (9.8)	3 (5.9)	10 (19.6)	17 (33.3)	19 (37.3)	4 (7.8)	7 (13.7)	4 (7.8)	13 (25.5)	19 (37.3)	6 (11.8)	4 (7.8)	9 (17.6)
Pain	33 (64.7)	9 (17.6)	3 (5.9)	1 (2.0)	5 (9.8)	28 (54.9)	9 (17.6)	7 (13.7)	3 (5.9)	4 (7.8)	27 (52.9)	13 (25.5)	6 (11.8)	3 (5.9)	2 (3.9)
Sleep	26 (51.0)	10 (19.6)	6 (11.8)	3 (5.9)	6 (11.8)	23 (45.1)	8 (15.7)	8 (15.7)	4 (7.8)	8 (15.7)	20 (39.2)	11 (21.6)	11 (21.6)	3 (5.9)	6 (11.8)
Daily routine	29 (56.9)	8 (15.7)	4 (7.8)	4 (7.8)	6 (11.8)	24 (47.1)	15 (29.4)	7 (13.7)	3 (5.9)	2 (3.9)	27 (52.9)	12 (23.5)	8 (15.7)	3 (5.9)	1 (2.0)
Work	34 (66.7)	7 (13.7)	6 (11.8)	1 (2.0)	3 (5.9)	33 (64.7)	10 (19.6)	5 (9.8)	3 (5.9)	-	32 (62.7)	11 (21.6)	4 (7.8)	2 (3.9)	2 (3.9)
Able to join activities	30 (58.8)	6 (11.8)	5 (9.8)	4 (7.8)	6 (11.8)	24 (47.1)	8 (15.7)	6 (11.8)	8 (15.7)	5 (9.8)	20 (39.2)	10 (19.6)	7 (13.7)	8 (15.7)	6 (11.8)

* Level 1: No, Level 2: A little bit, Level 3: A bit, Level 4: Quite a lot, Level 5 Very

For peer review only - http://bmjopen.bmj.com/site/about/guidelines.xhtml

Appendix 7: Sensitivity analyses

LEGO®-based therapy vs. usual support	Incremental costs (£), (95% CI)	Incremental QALYs (95% CI)	ICER (£/QALY gained), (95% CI)
Scenario 1: Complete case analysis from NHS perspective	-1,280 (-4,578 to 2,081)	0.011 (-0.017 to 0.040)	Dominant
Scenario 2: CUA from NHS and education perspective	-511 (-1,452 to 392)	0.009 (-0.008 to 0.028)	Dominant
Scenario 3: CUA from societal perspective	-376 (-1,377 to 595)	0.009 (-0.008, 0.028)	Dominant
Scenario 4: Assume outcomes were measured using CHU-9D	-246 (-719 to 246)	0.029 (0.009 to 0.049)	Dominant

For peer review only

For peer review only - http://bmjopen.bmj.com/site/about/guidelines.xhtml

CHEERS Checklist

Items to include when reporting economic evaluations of health interventions

The ISPOR CHEERS Task Force Report, Consolidated Health Economic Evaluation Reporting Standards (CHEERS)—Explanation and Elaboration: A Report of the ISPOR Health Economic Evaluations Publication Guidelines Good Reporting Practices Task Force, provides examples and further discussion of the 24-item CHEERS Checklist and the CHEERS Statement. It may be accessed via the Value in Health or via the ISPOR Health Economic Evaluation Publication Guidelines – CHEERS: Good Reporting Practices webpage: http://www.ispor.org/TaskForces/EconomicPubGuidelines.asp

**Section/item**	**Item No**	**Recommendation**	**Reported on page No/ line No**
**Title and abstract**			
Title	1	Identify the study as an economic evaluation or use more specific terms such as “cost-effectiveness analysis”, and describe the interventions compared.	Page 1
Abstract	2	Provide a structured summary of objectives, perspective, setting, methods (including study design and inputs), results (including base case and uncertainty analyses), and conclusions.	Page 2
**Introduction**			
Background and objectives	3	Provide an explicit statement of the broader context for the study. Present the study question and its relevance for health policy or practice decisions.	Page 4, Section 1
**Methods**			
Target population and subgroups	4	Describe characteristics of the base case population and subgroups analysed, including why they were chosen.	Section 2.1
Setting and location	5	State relevant aspects of the system(s) in which the decision(s) need(s) to be made.	Section 2.1
Study perspective	6	Describe the perspective of the study and relate this to the costs being evaluated.	Section 2.4
Comparators	7	Describe the interventions or strategies being compared and state why they were chosen.	Section 2.1
Time horizon	8	State the time horizon(s) over which costs and consequences are being evaluated and say why	Section 2.4

For peer review only

		appropriate.	
Discount rate	9	Report the choice of discount rate(s) used for costs and outcomes and say why appropriate.	Section 2.4
Choice of health outcomes	10	Describe what outcomes were used as the measure(s) of benefit in the evaluation and their relevance for the type of analysis performed.	Sections 2.3 and 2.4
Measurement of effectiveness	11a	Single study-based estimates: Describe fully the design features of the single effectiveness study and why the single study was a sufficient source of clinical effectiveness data.	Section 2.1
	11b	Synthesis-based estimates: Describe fully the methods used for identification of included studies and synthesis of clinical effectiveness data.	NA
Measurement and valuation of preference based outcomes	12	If applicable, describe the population and methods used to elicit preferences for outcomes.	Section 2.3
Estimating resources and costs	13a	*Single study-based economic evaluation*: Describe approaches used to estimate resource use associated with the alternative interventions. Describe primary or secondary research methods for valuing each resource item in terms of its unit cost. Describe any adjustments made to approximate to opportunity costs.	Section 2.4
	13b	*Model-based economic evaluation*: Describe approaches and data sources used to estimate resource use associated with model health states. Describe primary or secondary research methods for valuing each resource item in terms of its unit cost. Describe any adjustments made to approximate to opportunity costs.	NA
Currency, price date, and conversion	14	Report the dates of the estimated resource quantities and unit costs. Describe methods for adjusting estimated unit costs to the year of reported costs if necessary. Describe methods for converting costs into a common currency base and the exchange rate.	Section 2.4
Choice of model	15	Describe and give reasons for the specific type of decision-analytical model used. Providing a figure to show model structure is strongly recommended.	NA

For peer review only

Assumptions	16	Describe all structural or other assumptions underpinning the decision-analytical model.	NA
Analytical methods	17	Describe all analytical methods supporting the evaluation. This could include methods for dealing with skewed, missing, or censored data; extrapolation methods; methods for pooling data; approaches to validate or make adjustments (such as half cycle corrections) to a model; and methods for handling population heterogeneity and uncertainty.	Sections 2.5 and 2.6
Results			
Study parameters	18	Report the values, ranges, references, and, if used, probability distributions for all parameters. Report reasons or sources for distributions used to represent uncertainty where appropriate. Providing a table to show the input values is strongly recommended.	NA
Incremental costs and outcomes	19	For each intervention, report mean values for the main categories of estimated costs and outcomes of interest, as well as mean differences between the comparator groups. If applicable, report incremental cost-effectiveness ratios.	Sections 3.2, 3.3 and 3.4
Characterising uncertainty	20a	*Single study-based economic evaluation*: Describe the effects of sampling uncertainty for the estimated incremental cost and incremental effectiveness parameters, together with the impact of methodological assumptions (such as discount rate, study perspective).	Section 3.4 and 3.5
	20b	*Model-based economic evaluation*: Describe the effects on the results of uncertainty for all input parameters, and uncertainty related to the structure of the model and assumptions.	NA
Characterising heterogeneity	21	If applicable, report differences in costs, outcomes, or cost- effectiveness that can be explained by variations between subgroups of patients with different baseline characteristics or other observed variability in effects that are not reducible by more information.	NA
**Discussion**			
Study findings, limitations,	22	Summarise key study findings and describe how they support the conclusions reached. Discuss limitations	Section 4

For peer review only

generalisability, and current knowledge		and the generalisability of the findings and how the findings fit with current knowledge.	
**Other**			
Source of funding	23	Describe how the study was funded and the role of the funder in the identification, design, conduct, and reporting of the analysis. Describe other non-monetary sources of support.	Page 17
Conflicts of interest	24	Describe any potential for conflict of interest of study contributors in accordance with journal policy. In the absence of a journal policy, we recommend authors comply with International Committee of Medical Journal Editors recommendations.	Page 17

For consistency, the CHEERS Statement checklist format is based on the format of the CONSORT statement checklist

The ISPOR CHEERS Task Force Report provides examples and further discussion of the 24-item CHEERS Checklist and the CHEERS Statement. It may be accessed via the Value in Health link or via the ISPOR Health Economic Evaluation Publication Guidelines – CHEERS: Good Reporting Practices webpage: http://www.ispor.org/TaskForces/EconomicPubGuidelines.asp

The citation for the CHEERS Task Force Report is:
Husereau D, Drummond M, Petrou S, et al. Consolidated health economic evaluation reporting standards (CHEERS)—Explanation and elaboration: A report of the ISPOR health economic evaluations publication guidelines good reporting practices task force. Value Health 2013;16:231-50.

For peer review only
